# Identification of core carcinogenic elements based on the age-standardized mortality rate of lung cancer in Xuanwei Formation coal in China

**DOI:** 10.1038/s41598-023-49975-5

**Published:** 2024-01-02

**Authors:** Zailin Chen, Xianfeng Cheng, Xingyu Wang, Shijun Ni, Qiulian Yu, Junchun Hu

**Affiliations:** 1 Engineering Center of Yunnan Education Department for Health Geological Survey and Evaluation, Kunming, 652501 China; 2 Yunnan Land and Resources Vocational College, Kunming, 652501 China; 3 College of Earth Sciences, Chengdu University of Technology, Chengdu, 610059 China; 4 Coal Geology Prospecting Institute of Yunnan Province, Kunming, 650218 China

**Keywords:** Cancer, Environmental sciences, Health occupations

## Abstract

In this study, the core carcinogenic elements in Xuanwei Formation coal were identified. Thirty-one samples were collected based on the age-standardized mortality rate (ASMR) of lung cancer; Si, V, Cr, Co, Ni, As, Mo, Cd, Sb, Pb, and rare earth elements and yttrium (REYs) were analyzed and compared; multivariate statistical analyses (CA, PCA, and FDA) were performed; and comprehensive identification was carried out by combining multivariate statistical analyses with toxicology and mineralogy. The final results indicated that (1) the high-concentration Si, Ni, V, Cr, Co, and Cd in coal may have some potential carcinogenic risk. (2) The concentrations of Cr, Ni, As, Mo, Cd, and Pb meet the zoning characteristics of the ASMR, while the Si concentration is not completely consistent. (3) The REY distribution pattern in Longtan Formation coal is lower than that in Xuanwei Formation coal, indicating that the materials of these elements in coal are different. (5) The heatmap divides the sampling sites into two clusters and subtypes in accordance with carcinogenic zoning based on the ASMR. (6) PC1, PC2, and PC3 explain 62.629% of the total variance, identifying Co, Ni, As, Cd, Mo, Cr, and V. (7) Fisher discriminant analysis identifies Ni, Si, Cd, As, and Co based on the discriminant function. (8) Comprehensive identification reveals that Ni is the primary carcinogenic element, followed by Co, Cd, and Si in combination with toxicology. (9) The paragenesis of Si (nanoquartz), Ni, Co, and Cd is an interesting finding. In other words, carcinogenic elements Ni, Co, Cd, and Si and their paragenetic properties should receive more attention.

## Introduction

Lung cancer is one of the most common malignant tumors; it originates in the lung bronchial mucosa or glands and severely endangers human health and life^1–3^. Many countries have reported that the incidence rates and mortality from lung cancer have increased significantly in recent years, with higher incidences in males than females and higher incidences in urban areas than in rural areas. The age-standardized mortality rate (ASMR) values of lung cancer in 2018 were 27.1/10^5^ for men and 11.2/10^5^ for women, with an average of 19.2/10^5^^4^. The ASMRs of lung cancer in China were 27.91/10^5^, 40.32/10^5^ for men, 16.08/10^5^ for women, 30.33/10^5^ in cities, and 26.66/10^5^ in rural areas from 2004 to 2018^5^.

Northeast Yunnan (Xuanwei-Fuyuan) in China is an area that has high morbidity and mortality related to lung cancer^6^, and this area shows distinctive characteristics: (1) it is a typical rural area; (2) female incidence rates and mortality are high; (3) the pathological features are characterized by high proportions of lung adenocarcinoma^7–9^ and squamous cell carcinoma^10–13^; (4) the rates are four to eight times (4–8)^14^ the national average, and the incidence rate remains high^6,8^; and (5) areas with high morbidity from lung cancer are highly consistent with the development and application range of Xuanwei Formation coal^15–17^.

The burning of bituminous coal in Northeast Yunnan has been associated with the region's high reported incidence of lung cancer^18–20^. However, the specific cause is still a mystery^21–27^. Currently, the lung cancer incidence has shown no substantial relationship with smoking^28^ in this region. The polycyclic aromatic hydrocarbons (PAHs) present in coal^19–22,29^ may be a cause, but the putative dose‒response curves cannot fully explain the high morbidity from lung cancer^30–32^; in addition, poor geographical reproducibility is a major problem^33^. Nanoquartz particles^33,34^ may also be a cause, but some studies have shown that silica is not the main cause^19,24,35^. Therefore, these controversial nanoquartz particles are worth discussing again. In addition, the ASMR of lung cancer in Xuanwei-Fuyuan has geographical differences. It is urgent to explore the geographical differences of carcinogenic elements in coal, which is also the innovation of this paper.

Furthermore, according to current research results, high levels of potentially toxic elements exist in Northeast Yunnan in some coal mines^36–39^ and in the environment^17,27,40–44^; specifically, mainly Mn, Ti, Ni, V, Cr, Co, Cu, Sr, Zn, As, Mo, Cd, Pb, Cs, and Sb are present (Ni, V, Cr, Co, As, Cd, Mo, Pb, and Sb are carcinogenic^15,45–48^, but due to spatial complexity, it is not clear which elements play decisive roles^49^). Rare earth elements and yttrium (REYs) (La, Ce, Pr, Nd, Sm, Eu, Gd, Tb, Dy, Y, Ho, Er, Tm, Yb, Lu, and Y) are a group of elements that have similar geochemical properties^50,51^, including La, Ce, Pr, Nd, Sm, and Eu (light REYs (LREYs)) and Gd, Tb, Dy, Y, Ho, Er, Tm, Yb, and Lu (heavy REYs (HREYs))^52,53^, which are often used to identify rock characteristics and trace chemical processes^54^. To better assess carcinogenic elements, the behavior of REYs in coal must be understood^55^.

Multivariate statistical analyses^56,57^, including correlation analysis (CA)^58^, principal component analysis (PCA)^59,60^, and Fisher discriminant analysis (FDA)^61–63^, have been widely used as tools to identify sources and determine the main influential factors from compositional data. For example, Jin et al.^56^ applied PCA and CA to identify potential sources in soil and dust at children's playgrounds in Beijing; Ranjbar et al.^60^ utilized CA to establish the relationship between variables and PCA to reduce the dataset to several determining factors; He et al.^62^ used PCA and FDA to model, assess, and classify ecological and environmental quality and the impacts of coal mining; Bi et al.^61^ utilized the FDA model for mine water inrush sources. Hence, these methods can be used to reveal core carcinogenic elements in combination with toxicology.

In this study, the concentration and mineralogical characteristics of Si, Ni, V, Cr, Co, As, Mo, Cd, Pb, Sb, and REYs in coal were obtained to achieve the following objectives: (1) to understand the general characteristics of carcinogenic elements; (2) to understand the geographical differences in carcinogenic elements and REYs; (3) to identify the core carcinogenic elements; and (4) to fully explore carcinogenic information.

## Methodology

### Study area

Xuanwei city is located in the Wumeng Mountains in northeastern Yunnan Province, China. The geographical coordinates are 103° 35′ 30″ to 104° 40′ 50″ N and 25° 53′ 30″ to 26° 44′ 50″ E, with a total area of 6069.88 km^2^ and a population of 1.53 × 10^6^ (at the end of 2015). The production activities are mainly agricultural. Xuanwei city is one of the main coal production bases in Yunnan Province and has many small coal mines. The reported and predicted coal resources are 3.85 × 10^9^ tons, and the raw coal output is 2.7 × 10^6^ tons/year^16^.

Fuyuan County is located in the Wumeng Mountains in northeastern Yunnan Province. The geographical coordinates are 25° to 25°58' N and 103°58' to 104°49' E, with a total area of 3251 km^2^ and a population of 0.83 × 10^6^ (at the end of 2017). The economy is dominated by agricultural production (the agricultural population accounts for 92.93%), the industrial foundation is weak, and coal resources are abundant. The geological reserves of coal are 14.102 × 10^9^ tons, the reported reserves are 6.457 × 10^9^ tons, and the reported reserves of anthracite are 3.88 × 10^9^ tons. The raw coal output is more than 5 × 10^6^ tons/year^16^.

**Figure 1:**
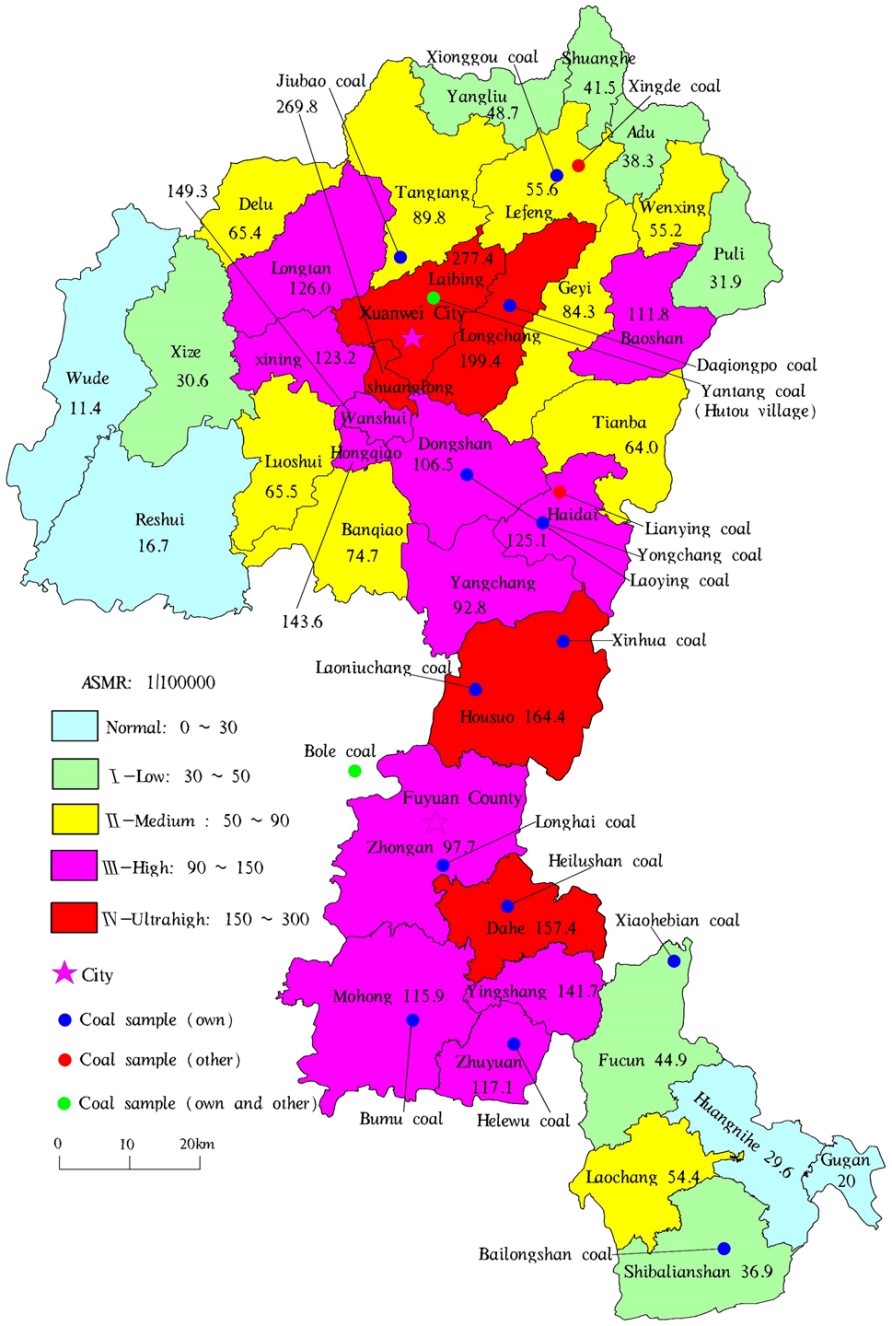
Geographical distribution of lung cancer ASMRs and coal sampling^6,8,66^.

Recent research shows that higher morbidity and mortality related to lung cancer exist in the entire coal-producing (burning) area of northeast Yunnan (Xuanwei-Fuyuan), and the problem remains serious and complex^6,41,64,65^. Hence, the geographical distribution of the ASMRs of lung cancer (Fig. 1) was plotted based on the latest data^6,8,66^. In this study, villages and towns were divided into five categories (normal, low, medium, high, and ultrahigh); among them, normal areas had no development or use of coal mines. The ASMRs of lung cancer in most villages and towns (Table S13) (II, III, and IV) were more than two times higher than that in China (27.91/10^5^)^5^, and those in some areas were more than four to eight times the national rate, with distinct geographical differences. Furthermore, the ASMR of lung cancer in zone I was low and could be used as excellent data for comparison. Hence, zones I, II, III, and IV were the focus of this paper.

### Sampling and analysis

#### Sample collection

Thirty-one (31) coal samples were collected based on the ASMR of lung cancer (Table S14) to explore the carcinogenic information carried in the coal samples^15^; information for other samples was compiled from the literature (some samples were from discontinued coal mines) (Tables S1, S2). Among them, the Xiaohebian and Bailongshan coals belong to the Longtan Formation, which is used for comparison. The coals were collected in sample bags, transported to the laboratory, ground, passed through a 200-mesh sieve, and prepared for use.

#### Analysis and quality control

##### Major elements

SiO_2_ was measured by an APL ADVANTXP + X instrument (X-ray fluorescence spectroscopy, 200 mesh)^15,52^, and then SiO_2_ (%) was changed to Si (mg/kg). The steps were as follows: (1) the organic matter was removed and then analyzed (1.0000 g of samples were thrown into a platinum crucible (5% Au + 95% Pt), whereupon the sample was placed in a muffle furnace (650 °C with the temperature raised for 1 h)); (2) the glass sample (dry sample) was made with a mixed flux (dehydrating agents NH_4_NO_3_ and LiBr and melting agent Li_2_B_4_O_7_ + LiBO_2_ + LiF); (3) finally, the glass sample was measured by X-ray fluorescence (XRF). The test accuracy (TA < 1%) and the detection limit (DL < 0.09%) met the requirements. The quality control experiment was considered satisfactory (RSD < 5%).

##### Trace elements

Trace elements were digested by a high-temperature closed digestion method^67^, and the concentrations were determined by inductively coupled plasma–mass spectrometry (ICP‒MS). The digestion steps, analytical steps, and quality control were based on our previous study^15,52^. The steps were as follows: (1) 0.10000 g of samples and 2 drops of ultrapure water were placed into a numbered 25 ml polytetrafluoroethylene liner (PTFEL) ((including 1 blank and 3 standard samples (GSS-1, GSS-3, and GSS-4)); (2) 2 ml HNO_3_ and 1 ml HF were added into the above PTFEL and placed into the digestion tank (DK); (3) the DK was tightened and placed in the oven (100 °C for 1 h, and 180 °C for 29 h); (4) the PTFEL was taken out from DK and then placed on an electric heating plate (130 °C, steamed dry); (5) the residual HF was removed (1 ml HNO_3_ was added and then steamed at 130 °C twice); (6) a standard sample (1 ml 1000 ng/ml Rh internal standard) was added to continue digestion (1 ml HNO_3_ and 5 ml ultrapure water were added to the PTFEL, and the DK was tightened and placed in the oven (130 °C for 1 h, and 140 °C for 4 h)); (7) dilution and filtration were performed (1 ml HNO_3_ was added, the volume was fixed to 10 ml with ultrapure water, and the sample was filtered with a 0.45 μM filtration membrane); (8) dilution was performed and determined by ICP‒MS (1 ml of solution was pipetted into a centrifuge tube and diluted to 10 ml with ultrapure water). The quality control met the requirements (RSD < 5%).

##### Optical microscopy

The thin and polished sections of coal were processed to observe the occurrence state of silicon by a polarizing microscope^37,68^ (Leica DM2700P and Jiangnan XPL-2) with reflected light and transmitted light^15^, and the magnifications were adjusted as needed (50–800 times).

##### Scanning electron microscopy (SEM)

The coal samples were processed and analyzed by a Hitachi S4800 (field emission scanning electron microscope)^52^, and the accuracy met the analytical and testing requirements (component analysis < 0.01%). First, the test bench and ceramic scissors (a degreasing cotton ball dipped in 95% ethanol) were wiped, and the sample was placed on a test bench containing conductive tape. Second, Au was sprayed on the sample to improve its conductivity. Afterward, the test bench was transferred to the scanning electron microscope compartment, and the microscopic morphology of the particles was tested.

##### Electron probe microanalysis (EMPA)

The quantitative analysis of in situ elements (Si, Ni, Co, and Cd) was completed by using an electron probe microanalyzer (JXA-8230 of JEOL)^69^. The analysis was completed using a JEOL JXA-8230 instrument, the voltage and current were 15 kV and 50 nA, the peak analysis time of Si, Ni, Co, and Cd was 30 ms, and the background analysis time was 30 ms.

### Identification of core carcinogenic elements

#### Correlation analysis (CA)

CA is used to comprehend the degree of resemblance and evaluate the relationships between carcinogenic elements and sources^70^. Furthermore, the latest research proposes heatmaps of correlation coefficients to exclude the dependency on variable variability^71,72^. In this study, the core carcinogenic elements were identified by analyzing the correlation between carcinogenic elements and REYs.

#### Principal component analysis (PCA)

Principal component analysis (PCA) aims to transform a set of potentially relevant variables into a set of linearly uncorrelated new variables through orthogonal transformation, which can retain the original information within the expressed information. Principal component analysis data processing plays a role in effectively eliminating correlations among high-dimensional data^73^, reducing the data dimensions, and simplifying the data structure.

#### Fisher discriminant analysis (FDA)

The Fisher discriminant method (the description is in the supplementary data S1) was proposed in 1936^74^, and it has no specific requirements for the overall distribution. In addition, it is a linear discriminant method^75,76^ that can discriminate among a small number of samples. It projects high-dimensional data points^77^ into low-dimensional space (one-dimensional straight line)^61^ so that the data points can become denser, and this can overcome the "curse of dimensionality" caused by high dimensionality. The principle of projection is to separate the population^78^ as much as possible, determine the discriminant analysis function according to the principle of maximum distance^79,80^ between classes and minimum distance within classes, and then classify and distinguish the new samples.

### Statistical analysis

The range of the elements, standard deviation (SD), median, mean, skewness, kurtosis, coefficient of variation (CV) and log_10_(x + 1) (Si: log_10_(x/1000 + 1)) functions were calculated via Microsoft Excel 2019^52^. As a note, log-transformation of each element is sufficient to put the data into normal distribution^60,72,81^ and thus meets the requirements of data processing. Statistical tests, including CA, PCA, and FDA, were performed using SPSS 25. The heatmap was implemented using R version 4.2.0.

### Ethical approval

All authors have read, understood, and complied as applicable with the statement on "Ethical responsibilities of Authors" as found in the Instructions for Authors.

## Results

### Elemental concentrations

#### Carcinogenic element concentrations

The concentrations of the investigated elements are listed in Tables S3 and S4, and the descriptive statistics for each in the Xuanwei Formation coal are shown in Table 1. The numerical value of each carcinogenic element exhibits a wide range. The mean concentrations of carcinogenic elements are 108,035 ± 40,748 mg/kg for Si, 101.45 ± 68.021 mg/kg for V, 35.81 ± 21.54 mg/kg for Cr, 23.29 ± 7.01 mg/kg for Co, 31.48 ± 11.70 mg/kg for Ni, 3.31 ± 4.86 mg/kg for As, 2.06 ± 1.42 mg/kg for Mo, 0.86 ± 0.75 mg/kg for Cd, 0.62 ± 0.63 mg/kg for Sb, and 15.13 ± 7.39 mg/kg for Pb.

**Table 1:** Statistical results of the carcinogenic element concentrations of Xuanwei Formation coal (mg/kg).

	Si	V	Cr	Co	Ni	As	Mo	Cd	Sb	Pb
Max	195,067	328.33	94.74	46.60	69.75	30.00	6.67	2.90	3.39	44.79
Min	40,413	15.12	9.00	8.45	13.78	0.34	0.27	0.05	0.06	2.99
Median	114,380	85.14	32.42	24.00	29.55	2.51	1.78	0.62	0.46	14.42
Mean	108,035	101.45	35.81	23.29	31.48	3.31	2.06	0.86	0.62	15.13
SD	40,748	68.02	21.54	7.01	11.70	4.86	1.42	0.75	0.63	7.39
Kurtosis	− 0.40	2.34	0.94	2.62	3.14	26.70	2.66	1.29	9.68	6.71
CV (%)	0.38	67.05	60.14	30.10	37.18	146.96	69.15	87.33	101.99	48.85
Skewness	0.09	1.37	1.09	0.62	1.51	4.89	1.53	1.44	2.64	1.85
Chinese coal	39,527	35.10	15.40	7.08	13.70	3.79	3.08	0.25	0.84	15.10
I-(low)	56,560	110.12	26.41	14.79	15.36	4.30	2.51	0.31	0.53	10.30

*Max* maximum; mean, *SD* standard deviation, *CV (%)* coefficient of variance.

The concentrations of Si, V, Cr, Ni, Co, As, Mo, Cd, Pb, and Sb are approximately 2.73, 2.89, 2.33, 3.29, 2.30, 0.87, 0.67, 3.44, 0.74, and 1.00 times the corresponding coal concentrations in China^37^, and the concentrations of Si, Ni, Cr, Co, Cd, Sb, and Pb are higher than those in Longtan Formation coal (I-(low)), indicating that Si, Cr, Co, Ni, and Cd may have some potential carcinogenic risk. Therefore, Si, Cr, Co, Ni, and Cd are given more attention in the following discussion.

#### Carcinogenic element comparison

According to a comparison of zones, the concentrations of Cr, Ni, As, Mo, Cd, and Pb in the coal of zone IV are higher than those in zones II and III (Table S15), indicating that the carcinogenic elements in coal are different.

The SiO_2_ content of the Xuanwei Formation coal is more than twice the average value of Chinese coal and more than twice that of the adjacent Longtan Formation coal. It is mainly closely related to quartz, which may lead to a high content of quartz particles in the local environment and indoor air, increasing the risk of local residents being exposed to quartz particles and causing pneumoconiosis. However, it is not completely consistent with the zoning characteristics of the ASMR, suggesting that there may be other collaborating factors. The concentrations of Cr, Ni, and Cd not only exceed those of Chinese coal and Longtan Formation coal but also meet the zoning characteristics of the ASMR. These may be the root cause of the difference in the ASMR.

#### REY comparison

The REY geochemical distribution model can directly reflect the differences in coal. In this study, the REY distribution curve was plotted based on the upper continental crust (UCC)^82,83^. The REY distribution patterns in zones I, II, III, and IV are characterized by LREY enrichment, weak negative Ce anomalies, and weak negative Y anomalies^63^ (Fig. S1). The REY distribution pattern in zone I (Longtan Formation coal) is lower than those in zones II, III, and IV (Xuanwei Formation coal), and there are also significant differences within the Xuanwei Formation coal, indicating that the materials of these elements in coal are different. Previous studies have revealed that the source distance and weathering intensity of the Emeishan basalt are factors^15,84–86^ that constrain the differences in the concentrations of REYs and carcinogenic elements. La, Ce, Pr, Nd, Eu, Gd, Tb, Dy, and REYs in the coal of zone IV are significantly lower than those in zones II and III, indicating that these elements (Table S11) can become environmental geochemical indicators for studying coal toxicity.

The geochemical parameters of REYs can reflect their degree of enrichment and material sources (Table S12). The degree of HREY fractionation ((Gd/Yb)_N_) is highly consistent with the ASMR zoning of lung cancer (the smaller the fractionation degree is, the higher the ASMR) based on the UCC. Moreover, the smaller the LREY/HREY and REY are, the higher the ASMR, indicating that these parameters can also be used as important indicators for predicting the carcinogenic elements present in Xuanwei Formation coal.

### Core carcinogenic element identification in the Xuanwei Formation coal

#### Core carcinogenic element identification (CA)

**Figure 2:**
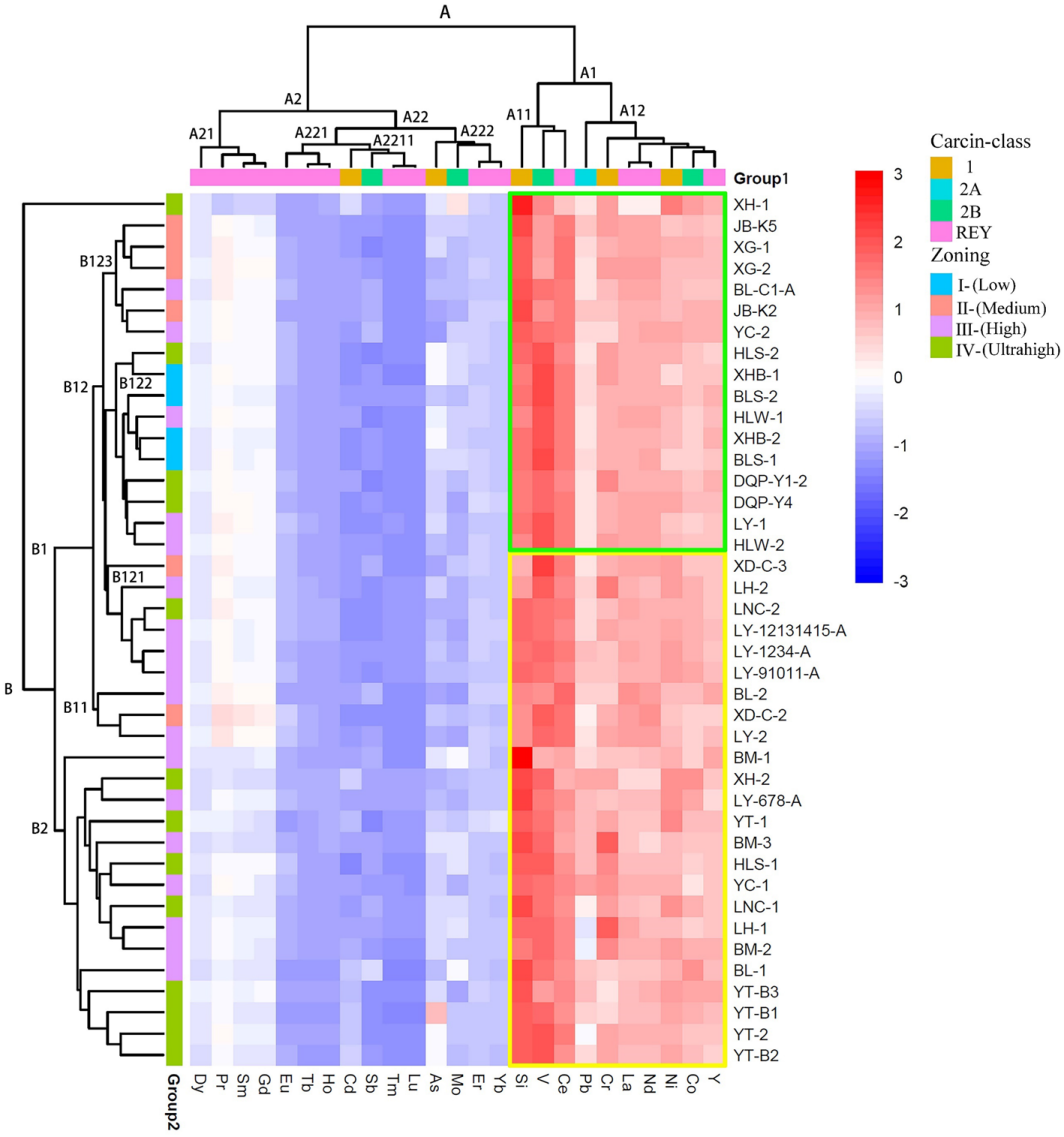
Heatmap of the correlation between sampling sites and studied elements.

Correlation analysis (including cluster analysis) was performed to determine the relationship between 25 elements (Si, Ni, V, Cr, Co, As, Mo, Cd, Sb, Pb, and REYs) and sampling sites (Fig. 1, Tables S18, S19, S20, S21) through heatmapping (Fig. 2). The right vertical dendrogram presents the clustering of the sampling sites (ASMRs of lung cancer zones). The horizontal dendrogram symbolizes the clustering of carcinogenic elements and REYs according to their similarities (the clustering basis for rows and columns that was chosen was “manhattan”, and the clustering method that was chosen was “mcquitty”).

The vertical tree graph on the right side of the correlation heatmap displays the clustering of sampling points, while the horizontal tree graph displays the clustering of elements. Overall, the carcinogenic elements in the study area can be divided into two clusters. Cluster A22 consists of Cd, Sb, As, and Mo, with a negative correlation with a high ASMR of lung cancer; among them, Cd and Sb (A2211) is a subtype indicating their similar geochemical behavior, probably with sulfur (S) compounds, while another subtype, As and Mo (A222), represents hydrothermal influence^87^. Cluster A1 consists of Si, V, Pb, Cr, Ni, and Co, with a positive correlation with a high ASMR of lung cancer; subtype A11 consists of Si, V, and Ce, indicating that the influence of sea water on coal seams during coalification due to Ce anomalies is controlled by the seawater content^88–90^; and subtype A12 consists of Pb, Cr, La, Nd, Ni, Co, and Y, indicating that carcinogenic elements inside come from the weathering of the Emeishan basalt^91–93^ during coalification. This phenomenon also exists objectively in other coal areas worldwide^94–99^. The heatmap divides sampling sites into two clusters and several subtypes in accordance with carcinogenic zoning based on the ASMR, indicating the effectiveness of ASMR partitioning in this paper. In addition, they are elements with significant concentration centers in Xuanwei and Fuyuan^100^.

Hence, Si, V, Cr, Co, and Ni are identified; Pb has a weak correlation; and abnormalities in Y and Ce^68,101^ can be important indicators for predicting carcinogenic elements in coal.

#### Core carcinogenic element identification (PCA)

To verify the above conclusions, a PCA of 10 carcinogenic elements in coal was performed. All factors were obtained with eigenvalues > 1^60^ and then rotated using the varimax method in SPSS 25 (Kaiser normalization), and finally, the rotation converged in 5 iterations^102^. The PCA results revealed five factors (82.633% of the total variance (TV)), and PC5 was used as a reference only for its eigenvalues < 1. The first three principal components (PCs) explain 27.444% (PC1), 18.664% (PC2), and 16.521% (PC3) of the total variance in carcinogenic element concentrations.

PCA reduced the dataset to^60,103^ major factors to explore the source of carcinogenic elements detected in coal. Moreover, the varimax rotation method^104^, Kaiser‒Meyer‒Olkin (KMO)^105^, and Bartlett’s sphericity test^106^ were used. In addition, the dataset was standardized and transformed using the log_10_(x + 1)-scale before PCA. The principal components (PC1, PC2, and PC3), loadings of variables (LV), eigenvalues (EV), and their respective variances (RV) are displayed in Table S16. In our study, three PCs were extracted, accounting for 62.629% of the total variance, revealing the main carcinogenic elements in Xuwanwei Formation coal. The factor loadings were divided into "strong", "medium", and "weak" in terms of the absolute loading values of > 0.75, 0.75–0.50, and 0.50–0.30, respectively.

PC1 consists of Ni and Co (Table S16), explains 27.444% of the TV and 2.744% of the EV, and has strong positive loadings of Co (0.893) and Ni (0.840). Considering their high concentrations and siderophile features, Ni and Co probably originated from the weathering of the Emeishan basalt^91–93,107^ during coalification, which is highly consistent with the CA results.

PC2 consists of As, Cd, and Mo (Table S16) and explains 18.664% of the TV and 1.866% of the EV. These carcinogenic elements are probably present due to the contribution of hydrothermal activity during coalification^36,97,108^.

PC3 consists of Cr and V (Table S16), explains 16.521% of the TV and 1.652% of the EV, and has strong positive loadings of Cr (0.862) and V (0.847). These carcinogenic elements are siderophile elements and originated from the weathering of the Emeishan basalt and the influence of sea water during coalification, which is highly consistent with CA.

Among them, PC1, PC2, and PC3 explain 62.629% of the total variance. Hence, Co, Ni, As, Cd, Mo, Cr, and V are identified.

#### Core carcinogenic element identification (FDA)

The Fisher discriminant function was calculated for four groups of samples (I, II, III, and IV). Table S5 shows that the significance probability of Co, Ni, As, Cd, and Si is less than 0.05 (rejecting the original hypothesis), indicating that the Co, Ni, As, Cd, and Si included in the discriminant function play a role in determining the correct classification. Therefore, Co, Ni, As, Cd, and Si were selected for Fisher discriminant analysis.

The significance test results of the discriminant function show that (Table S6) the Wilks' lambda value of the function from 1 to 3 is 0.320, the chi-squared value is 40.460, the degree of freedom (Df) is 15, and the significance probability is 0.000. The discriminant function has reference significance.

Table S7 shows that the value of the box's M is 114.604, which meets the calculation requirements (> 0.05). Consequently, all kinds of covariance matrices were considered equal and met the requirements for the test results (Yang et al. 2017). At the same time, the significance probability^109^ of the F test is less than 0.05, indicating that the error probability of the discriminant function is small.

The variance percentage can be used as an interpretation of the discriminant equation. The variance percentage of discriminant function 1 is 83.3% (Table S8), so this function could discriminate most samples. The structure matrix represents the intragroup correlations between the discriminant variable and the standardized canonical discriminant function (Table S9). According to the absolute size of the intrafunction correlation, it consists of Ni (0.722), Si (0.515), Cd (0.416), As (0.107), and Co (0.471). Based on the absolute size of the intrafunction correlation, Ni shows the largest correlation, followed by Co. The function and combination characteristics serve as important bases for identifying the carcinogenic elements Ni, Si, Cd, As, and Co.

**Figure 3:**
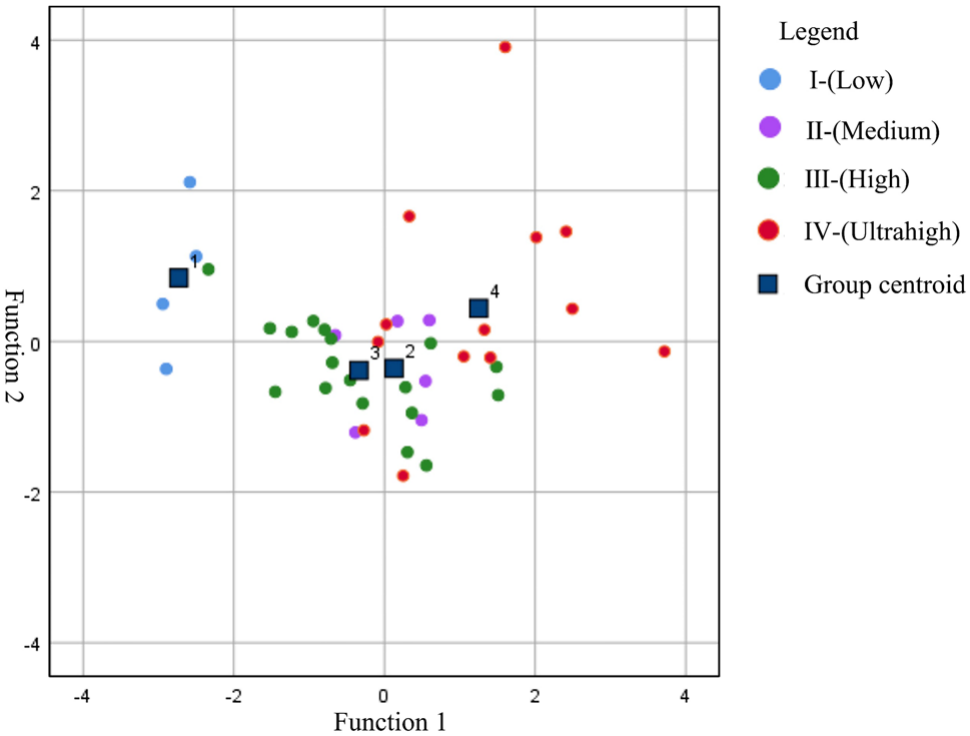
Comprehensive results of the discriminant function.

According to the coefficients and constant terms of discriminant function 1, function 2, and function 3 (Table S10), two groups of functions and comprehensive results were obtained (Fig. 3), which clearly distinguishes each group:

1 $$ {\text{Function 1:}}\,\,\,\,\,{\text{Y1}} = - 0.{\text{821Co}} + {6}.{7}0{\text{5Ni}} - 0.{\text{774As}} + {2}.{\text{443Cd}} + {3}.{\text{596Si}} - {15}.{938} $$

2 $$ {\text{Function 2}}:\,\,{\text{Y}}2 = 0.396{\text{Co}} - 0.974{\text{Ni}} + 4.350{\text{As}} - 0.132{\text{Cd}} - 0.420{\text{Si}} - 0.62 $$

3 $$ {\text{Function 3:}}\,\,{\text{Y3}} = {9}.{\text{851Co}} - {4}.{\text{862Ni}} + {1}.{1}0{\text{3As}} - {3}.{\text{756Cd}} + 0.{4}0{\text{4Si}} - {6}.{726} $$

The comprehensive result map of the Fisher discriminant function of carcinogenic elements established in this study can be used to easily and quickly distinguish the carcinogenic characteristics of the coal in the study area and provide a quantitative method for deepening the understanding of the environmental geochemical characteristics of coal. However, a single mathematical statistical discrimination procedure cannot be used as a sufficient condition for establishing coal carcinogenic characteristics, and other indicators need to be used to confirm the findings.

## Discussion

The CA, PCA, and FDA had high discrimination accuracy. However, if the toxicological characteristics of carcinogenic elements and the mineralogical characteristics of silicon are not considered, the effect of some carcinogenic elements can be exaggerated, and the results of multivariate statistical analyses are distorted. Therefore, toxicology is also the focus of this paper.

### Toxicological characteristics of carcinogenic elements

Si, Ni, V, Cr, Co, As, Mo, Cd, Pb, and Sb were filtered out based on the findings of substantial previous scientific studies^17,27,33,41–44,110^. Nevertheless, the correlations between these carcinogenic elements and lung cancer as well as their toxicological characteristics^45,47,48^ need to be considered (Table S17).

Some important information can be obtained from Table S17: (1) crystalline silica, Cr^6+^, Ni, As, and Cd are classified as class I carcinogens; and (2) the toxicological characteristics of crystalline silica, Co, Ni, Cr^6+^, and Cd are connected with lung cancer (Table S17). However, the concentration of Cr^6+^ in local coal^111^ and the environment^102^ seems not to be the main cause. Hence, Si, Co, Ni, As, and Cd should be given more attention.

### Mineralogical characteristics of silicon

Amorphous silica is commonly present in nature and has little or no chronic adverse pulmonary effects^112–116^, such as in sedimentary rocks^117,118^, hot spring systems^119,120^, and soil^121^. Crystalline silica is a class I carcinogen^112^.

**Figure 4:**
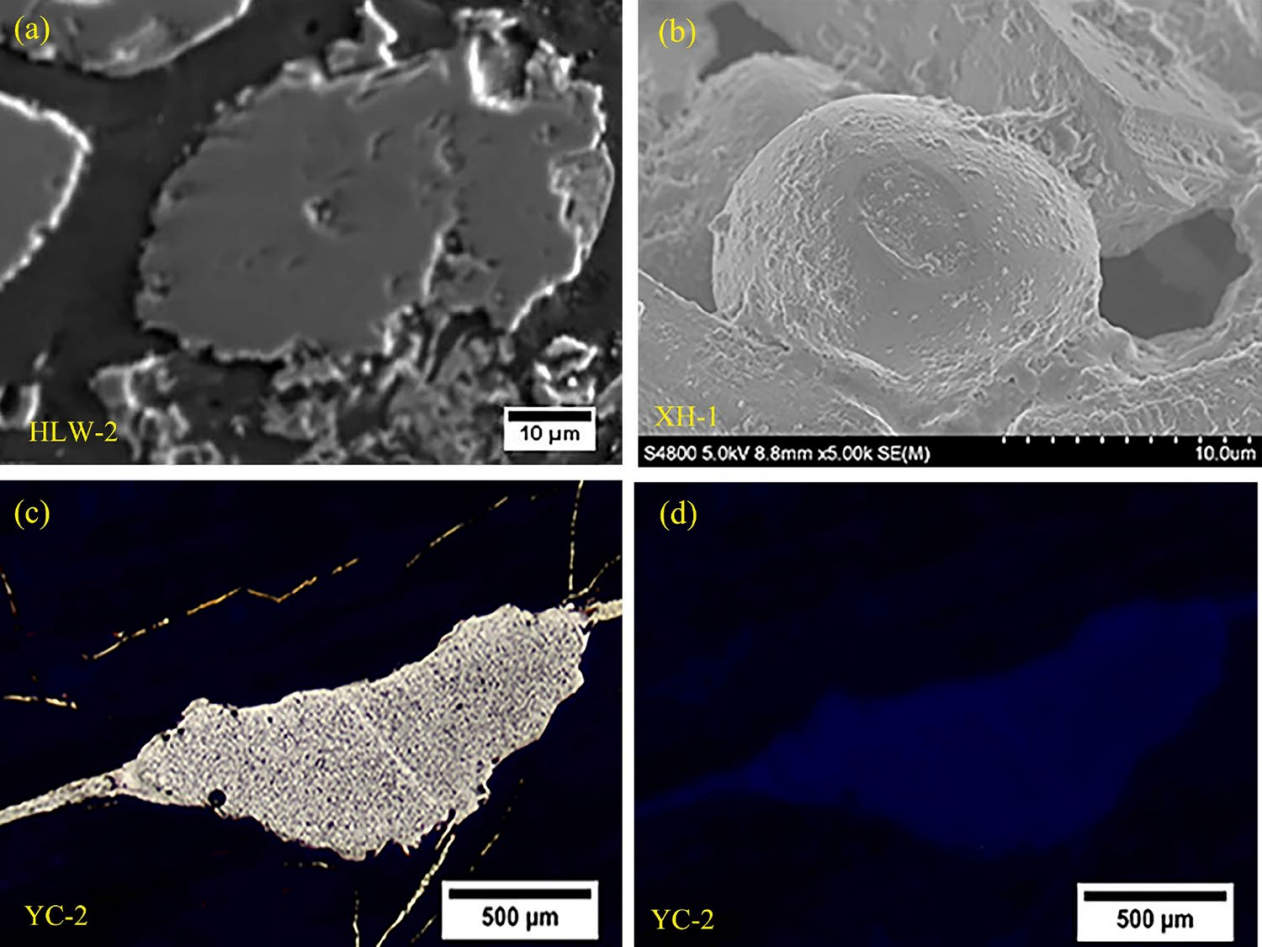
Occurrence state of authigenic silica. (**a**) Backscattered electron (BSE) image; (**b**) electron micrographs from silica nanoparticles; (**c**) plane-polarized light, amorphous silica; (**d**) cross-polarized light, complete extinction of amorphous silica.

The occurrence state of silicon in the coal of the Xuanwei Formation is mainly authigenic quartz^36,37^ (Fig. 4), except for terrigenous clastic quartz and pyroclastic quartz. In addition, respirable silica refers to silica particles less than 10 μm in diameter^122^, widely existing in the Xuanwei Formation coal (Fig. 4b). Therefore, we should pay attention to the crystalline quartz in coal and the conditions under which it is converted into respirable quartz^122^.

### Comprehensive identification

We reduced Si, Ni, V, Cr, Co, As, Mo, Cd, Pb, and Sb to Si, Co, Ni, As, and Cd according to the toxicological characteristics. Furthermore, the concentration, CA, PCA, and FDA methods identified the carcinogenic elements. However, the core carcinogenic elements were still unclear. Therefore, it was necessary to combine concentration, CA, PCA, and FDA with toxicology and mineralogy to achieve comprehensive identification because each method has its limitations.

Table 2 shows that Ni was the primary core carcinogenic element, followed by Co, Cd, and Si, which was consistent with the conclusions of our previous research^102^. Hence, accurate and effective identification of core carcinogenic elements (Ni, Co, Cd, and Si) can support the development and use of local coal in the future.

**Table 2:** Comprehensive identification table.

Method	Si	V	Cr	Co	Ni	As	Mo	Cd	Sb	Pb
Concentration	√		√	√	√			√		
IARC classification	√			√	√			√		
CA	√	√	√	√	√					
PCA		√	√	√	√	√	√	√		
FDA	√			√	√	√		√		

**Figure 5:**
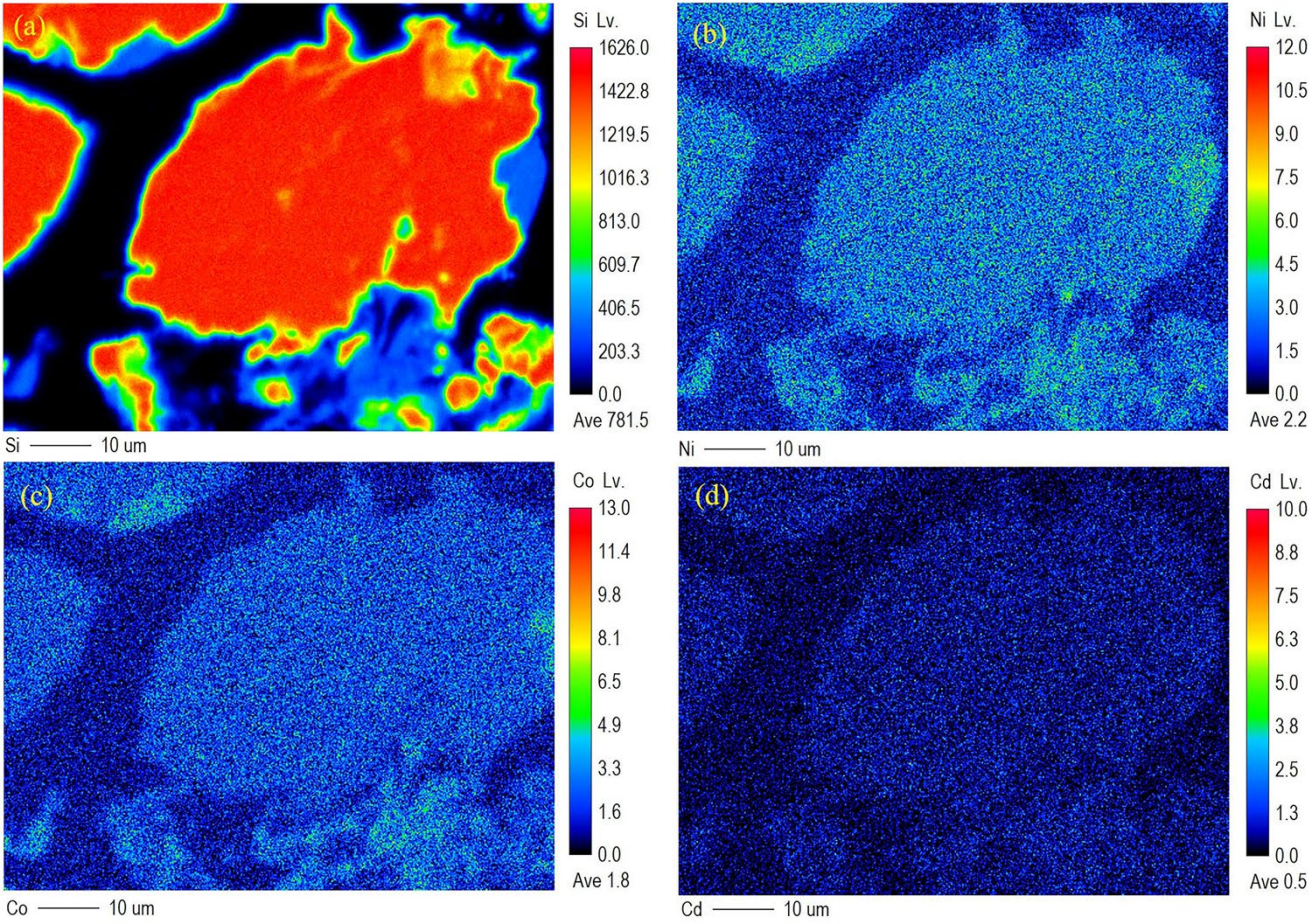
X-ray maps for the distribution of Si, Ni, Co, and Cd in coal (HLW-2) (Lv represents the signal strength level).

Interestingly, Si (nanoquartz), Ni, Co, and Cd are highly paragenetic (Fig. 5), consistent with the EDX spectrum of silica particles in air^24^, which seems to enhance the carcinogenic activity of local coal. More studies are needed to better comprehend the role of carcinogenic elements and crystalline quartz paragenesis in carcinogenesis in this area.

### Discussion on the causes of cancer risk

Quartz is a class I carcinogen, and its carcinogenic mechanism is that it leads to pulmonary fibrosis (pneumoconiosis, silicosis)^123–125^ after inhalation into the lungs and induces cancer. Pneumoconiosis related to coal miners is common worldwide^6,126^, and the Xuanwei Fuyuan area is no exception^109,127^. However, the incidence of lung cancer in Xuanwei and Fuyuan is not only among coal miners but also among those who use local coal for heating or cooking, indicating a synergistic effect of factors other than quartz. Li^128^ believes that residents in the Xuanwei Fuyuan area have been exposed to quartz particles and that the inflammatory response is the key factor leading to lung injury^88^. Quartz particles easily penetrate the bronchial epithelial cell membrane and enter the cell but cannot enter the nucleus. At present, most scientists believe that local nanoquartz particles are synergistic carcinogens and synergists^19,24,35^.

Exposure to nickel in the environment is related to human lung cancer and nasal cancer^104,129^, and research has shown that its triggering of DNA damage (cell cycle imbalance) is an important carcinogenic mechanism. In vitro and in vivo experiments show that nickel can produce reactive oxygen species by binding DNA, mediating DNA damage and inhibiting DNA repair^130,131^. Coal mines that have high Ni contents include the Candiota and Colchester low-grade coal mine in the Shenbei lignite in China and Brazil, respectively, and the Kosovo lignite mine in Serbia^37,96,98,99,132^. Notably, the lung cancer incidence in these areas is also worrisome^94,95,108^. The correlation between nickel and lung cancer is evident worldwide^133–135^. The carcinogenic mechanism of Ni in coal remains to be explored. Morbidity from lung cancer has been linked with exposure to high contents of nickel compounds^136,137^, including sulfidic, oxidic, water-soluble, and insoluble metallic nickel^138–141^. Among them, water-soluble nickel has a greater oral absorption and is the most important risk factor. Furthermore, studies have shown that a dose-related association^139^ of cumulative exposure to water-soluble nickel compounds could lead to lung cancer^139,142^. At present, a high nickel content and high nickel water solubility have been found in the PM_10_ of Hutou village (Fig. 1) from coal development and use, which probably supports this view. Epithelial-mesenchymal transitional lung injury is the mechanism of nickel-induced lung diseases^137,143^. Coincidentally, the pathological feature of lung cancer in Fuyuan and Xuanwei is the damage of epithelial cells, with a high proportion of squamous cell carcinoma^144^ and adenocarcinoma^10,12^. Nickel appears to be the most dangerous carcinogen in the local area.

Cobalt exposure in the environment is a recognized cause of human interstitial lung disease (which can later develop into diffuse pulmonary fibrosis)^145^, generally occurring in hard metal and bonded diamond tool industries. There have also been reports of interstitial lung disease related to coal mines, but further work is needed to determine whether it is related to cobalt in coal^146,147^.

Cadmium is a known human lung carcinogen, and its main carcinogenic mechanism is damage to lung epithelial cells^148^. In vitro studies have revealed possible toxicokinetic pathways, such as increased oxidative stress, changes in transcription factor activity and inhibition of DNA repair^149^. Particles with aerodynamic diameters less than 10 µm can be used as carriers of cadmium, which affects lung health. The increasing trend of lung cancer mortality related to Cd in coal has also been reported worldwide^150^. The pathological characteristics of lung cancer in Xuanwei Fuyuan are a high proportion of adenocarcinoma and squamous cell carcinoma^13^. From a pathological point of view, the lung cancer is due to the abnormal proliferation of adenoid epithelium and squamous epithelium, which may be related to Cd in atmospheric particles.

Therefore, this paper infers that the synergetic carcinogenic mechanism of nanoquartz particles (Si), Ni, Co and Cd in Xuanwei Formation coal is that "nanoquartz particles and Co damage lung tissue cells (inflammatory reaction), and Ni and Cd damage lung nuclei (mediate DNA damage and inhibit DNA repair)". This conclusion still needs further verification through medical experiments in the future.

## Conclusions

The current research results provide information on the characteristics of core carcinogenic elements in coal from the Xuanwei Formation. The results demonstrated the following:

(1) The concentrations of Si, Ni, V, Cr, Co, and Cd were higher than those in Chinese coal and Longtan Formation coal; (2) the heatmap of correlation identified Si, V, Cr, Co, and Ni; PCA identified Co, Ni, As, Cd, Mo, Cr, and V; FDA identified Ni, Si, Cd, As, and Co; (3) comprehensive identification revealed that Ni was the primary carcinogenic element, followed by Co, Cd, and Si in combination with toxicology; and (4) the paragenesis of Si (nanoquartz), Ni, Co, and Cd in coal may increase the possibility of carcinogenesis.

### Supplementary Information

Supplementary Information.

## Data Availability

All data generated or analyzed during this study are included in this published article [and its supplementary information files].
