# Supplementary material for: Identification of core carcinogenic elements based on the age-standardized mortality rate of lung cancer in Xuanwei Formation coal in China
Source: Sci Rep. 2024 Jan 2;14:232. doi: 10.1038/s41598-023-49975-5 (PMC10761687; doi:10.1038/s41598-023-49975-5)
Supplement: Supplementary file 1 — Supplementary Information. [file 41598_2023_49975_MOESM1_ESM.docx]

**Supplementary data**

Table S1. Geographical location and collected data with the number of samples.

| Coal name | East longitude | North latitude | Number of samples | Sources |
| --- | --- | --- | --- | --- |
| Xinde coal mine | 104°19'44"~  104°22' 59" | 26°28'46"~  26°30'18" | 6 | (Dai et al., 2014) |
| Xionggou coal mine | 104°19′26″~ 104°20′27″ | 26°28′46″~  26°30′45″ | 2 | This study |
| Yantang coal mine | 104°07'52"~  104°08'35" | 26°18'17"~  26°19'06" | 3+2 | (Dai et al., 2008; This study) |
| Jiubao coal mine | 104°11'33"~  104°12'43" | 26°24'25"~  26°25'56" | 2 | This study |
| Daqingpo coal mine | 104°19′12″~  104°20′28″ | 26°21′28″~  26°22′45″ | 2 | This study |
| Laoying coal mine | 104°15′49″~  104°16′27″ | 26°04′10″~  26°05′05″ | 2 | This study |
| Yongchang coal mine | 104°20'51"~  104°22'04" | 26°02'55"~  26°03'26" | 2 | This study |
| Lianying coal mine |  |  | 52 | (Zheng, 2018) |
| Bole coal mine | 104°06′15″~ 104°07′48″ | 25°46′53″~  25°48′24″ | 23+2 | (Li, 2015; This study) |
| Laoniuchang coal mine | 104°18′34″~  104°19′21″ | 25°49′30″~  25°50′17″ | 2 | This study |
| Longhai coal mine | 104º16′20″~  104º17′03″ | 25º37′45″~  25º38′31″ | 2 | This study |
| Xinhua Coal Mine | 104°22'47″~  104°23'37" | 25°49'17"~  25°49'44" | 2 | This study |
| Heilushan coal mine | 104°18′45″~  104°19′45″ | 25°35′03″~  25°35′54″ | 2 | This study |
| Bumu coal mine | 104°13′06″~  104°14′07″ | 25°28′09″~  25°29′22″ | 3 | This study |
| Helewu coal mine | 104°12′29″~  104°13′13″ | 25°19′35″~  25°20′17″ | 2 | This study |
| Xiaohebian coal mine | 104°30′45″~  104°32′27″ | 25°30′30″~  27°31′45″ | 2 | This study |
| Bailongshan coal mine | 104°34′~ 104°38′ | 25°10′~  25°14′ | 2 | This study |

**Reference:**

Dai, S., Tian, L., Chou, C. L., Zhou, Y., Zhang, M., Zhao, L., Ren, D. 2008. Mineralogical and compositional characteristics of Late Permian coals from an area of high lung cancer rate in Xuan Wei, Yunnan, China: Occurrence and origin of quartz and chamosite. Int. J. Coal. Geol., 76(4), 318-327. [https://doi.org/ 10.1016/j.coal.2008.09.001](https://doi.org/%2010.1016/j.coal.2008.09.001).

Dai, S., Li, T., Seredin, V. V., Ward, C. R., Hower, J. C., Zhou, Y., Zhao, C. 2014. Origin of minerals and elements in the Late Permian coals, tonsteins, and host rocks of the Xinde Mine, Xuanwei, eastern Yunnan, China. Int. J. Coal. Geol.,121, 53-78.

[https://doi.org/ 10.1016/j.coal.2013.11.001](https://doi.org/%2010.1016/j.coal.2013.11.001).

Li, X., 2015. Mineral matter Characteristic and sources of volcanic ash in the Late Permian coal-bearing strata from Xuanwei, eastern Yunnan. China University of mining and Technology (Beijing). (in Chinese). <http://cdmd.cnki.com.cn/Article/CDMD-11413-1015304620.htm>.

Zheng, X., 2018. Mineral Matter in Lopingian Coals from Eastern Yunnan Province and Its Response to the Regional Geological Evolution. China University of mining and Technology (Beijing). (in Chinese). http://cdmd.cnki.com.cn/Article/CDMD-11413-1018169282.htm.

Table S2. Sample name and sources from respective coal mine area.

| Coal name | Sample name | Sources |
| --- | --- | --- |
| Xinde coal mine | XD-C-2, XD-C-3 | (Dai et al., 2014) |
| Xionggou coal mine | XG-1, XG-2 | This study |
| Yantang coal mine | YT-1, YT-2 | This study |
|  | YT-B1, YT-B2, YT-B3 | (Dai et al., 2008) |
| Jiubao coal mine | JB-K2, JB-K5 | This study |
| Daqingpo coal mine | DQP-Y1-2, DQP-Y4 | This study |
| Laoying coal mine | LY-1, LY-2 | This study |
| Yongchang coal mine | YC-1, YC-2 | This study |
| Lianying coal mine | LY-1234-A, LY-678-A, LY-91011-A, LY-12131415-A | (Zheng, 2018) |
| Bole coal mine | BL-C1-A | (Li, 2015) |
|  | BL-1, BL-2 | This study |
| Laoniuchang coal mine | LNC-1, LNC-2 | This study |
| Longhai coal mine | BL-1, BL-2 | This study |
|  | BL-C1-A | This study |
| Xinhua Coal Mine | XH-1, XH-2 | This study |
| Heilushan coal mine | HLS-1, HLS-2 | This study |
| Bumu coal mine | BM-1, BM-2, BM-3 | This study |
| Helewu coal mine | HLW-1, HLW-2 | This study |
| Xiaohebian coal mine | XHB-1, XHB-2 | This study |
| Bailongshan coal mine | BLS-1, BLS-2 | This study |

**Reference:**

Dai, S., Tian, L., Chou, C. L., Zhou, Y., Zhang, M., Zhao, L., Ren, D. 2008. Mineralogical and compositional characteristics of Late Permian coals from an area of high lung cancer rate in Xuan Wei, Yunnan, China: Occurrence and origin of quartz and chamosite. Int. J. Coal. Geol., 76(4), 318-327. [https://doi.org/ 10.1016/j.coal.2008.09.001](https://doi.org/%2010.1016/j.coal.2008.09.001).

Dai, S., Li, T., Seredin, V. V., Ward, C. R., Hower, J. C., Zhou, Y., Zhao, C. 2014. Origin of minerals and elements in the Late Permian coals, tonsteins, and host rocks of the Xinde Mine, Xuanwei, eastern Yunnan, China. Int. J. Coal. Geol.,121, 53-78.

[https://doi.org/ 10.1016/j.coal.2013.11.001](https://doi.org/%2010.1016/j.coal.2013.11.001).

Li, X., 2015. Mineral matter Characteristic and sources of volcanic ash in the Late Permian coal-bearing strata from Xuanwei, eastern Yunnan. China University of mining and Technology (Beijing). (in Chinese). <http://cdmd.cnki.com.cn/Article/CDMD-11413-1015304620.htm>.

Zheng, X., 2018. Mineral Matter in Lopingian Coals from Eastern Yunnan Province and Its Response to the Regional Geological Evolution. China University of mining and Technology (Beijing). (in Chinese). <http://cdmd.cnki.com.cn/Article/CDMD-11413-1018169282.htm>.

**Table S3** Abundances of trace elements in Xuanwei Formation coal (mg/kg).

| Element | YT-1 | YT-2 | YT-B1 | YT-B2 | YT-B3 | JB-K5 | JB-K2 | DQP-Y1-2 | DQP-Y4 |
| --- | --- | --- | --- | --- | --- | --- | --- | --- | --- |
| Si | 155820 | 168233 | 128987 | 138973 | 83720 | 116480 | 127167 | 139860 | 104907 |
| V | 106.89 | 189.15 | 107 | 166 | 27 | 32.49 | 36.17 | 150.45 | 193.31 |
| Cr | 42.94 | 44.15 | 17 | 36 | 9 | 32.18 | 15.95 | 86 | 46.57 |
| Co | 27.78 | 24.08 | 31 | 28 | 24 | 19.97 | 21.35 | 29.02 | 36.09 |
| Ni | 69.75 | 41.47 | 41 | 40 | 27 | 18.74 | 19.55 | 38.43 | 63.34 |
| As | 3.17 | 7.88 | 30 | 7.2 | 2.9 | 2.51 | 0.76 | 3.1 | 4.21 |
| Mo | 2.51 | 2.44 | 2.7 | 1.7 | 0.84 | 2.23 | 1.2 | 1.78 | 1.39 |
| Cd | 1.65 | 2.41 | 2.9 | 2.5 | 1.4 | 0.43 | 0.74 | 1.14 | 0.71 |
| W | 0.93 | 1.02 | 0.7 | 0.81 | 0.59 | 0.82 | 7.23 | 0.88 | 12.04 |
| Pb | 30.93 | 7.26 | 19 | 16 | 18 | 18.32 | 14.79 | 15.16 | 18.29 |
| La | 24.01 | 39.18 | 26 | 28 | 18 | 27.59 | 21.79 | 44.11 | 53.32 |
| Ce | 50.99 | 89.62 | 56 | 51 | 38 | 59.9 | 49.94 | 87.55 | 120.64 |
| Pr | 5.97 | 9.71 | 7.2 | 6.5 | 5.1 | 7.05 | 5.87 | 11 | 13.19 |
| Nd | 22.2 | 36.98 | 27 | 25 | 20 | 27.6 | 23.55 | 45.34 | 53.16 |
| Sm | 4.44 | 6.6 | 5.7 | 4.6 | 4.6 | 5.82 | 4.97 | 8.48 | 10.9 |
| Eu | 0.64 | 1.17 | 1 | 1.2 | 0.82 | 0.89 | 0.89 | 2.21 | 1.81 |
| Gd | 4.06 | 6.7 | 5.1 | 4.1 | 4.7 | 5.12 | 5.15 | 7.91 | 10.59 |
| Tb | 0.82 | 0.97 | 0.85 | 0.62 | 0.76 | 0.73 | 0.79 | 1.14 | 1.39 |
| Dy | 5.37 | 5.02 | 5 | 3.4 | 4.3 | 3.69 | 4.05 | 6.15 | 7.01 |
| Y | 29.05 | 26.77 | 30 | 19 | 23 | 18.96 | 22.19 | 38.27 | 38.72 |
| Ho | 1.21 | 1.02 | 1 | 0.65 | 0.81 | 0.71 | 0.83 | 1.25 | 1.51 |
| Er | 3.78 | 2.84 | 3 | 1.9 | 2.2 | 2.02 | 2.37 | 3.39 | 4.61 |
| Tm | 0.64 | 0.39 | 0.43 | 0.28 | 0.32 | 0.3 | 0.34 | 0.47 | 0.65 |
| Yb | 4.45 | 2.5 | 2.8 | 1.8 | 2 | 1.79 | 2.29 | 2.67 | 4.49 |
| Lu | 0.59 | 0.37 | 0.4 | 0.26 | 0.29 | 0.26 | 0.35 | 0.39 | 0.63 |

- continued.

| Element | XG-1 | XG-2 | XD-C-2 | XD-C-3 | LY-1234-A | LY-678-A | LY-91011-A | LY-12131415-A |
| --- | --- | --- | --- | --- | --- | --- | --- | --- |
| Si | 126233 | 85120 | 140887 | 118673 | 139720 | 117647 | 106493 | 121427 |
| V | 40.42 | 35.64 | 255.5 | 328.33 | 74.75 | 50.2 | 63.8 | 86.96 |
| Cr | 26.99 | 48.99 | 51.88 | 36.4 | 15.99 | 12.84 | 15.48 | 31.69 |
| Co | 25.74 | 24.6 | 27.38 | 28.37 | 22.51 | 23.21 | 27.01 | 25.85 |
| Ni | 29.34 | 27.56 | 38.6 | 52.6 | 33.02 | 30.17 | 31.11 | 32.49 |
| As | 3.08 | 1.7 | 3.27 | 1.09 | 1.63 | 0.52 | 1.21 | 1.17 |
| Mo | 1.35 | 2.16 | 2.46 | 2.18 | 0.67 | 0.27 | 1.09 | 0.71 |
| Cd | 0.62 | 0.94 | 0.43 | 0.52 | 0.23 | 0.05 | 0.35 | 0.19 |
| W | 1.25 | 6.12 | 0.42 | 0.48 | 4.81 | 4.2 | 2.43 | 2.68 |
| Pb | 13.94 | 13.26 | 17.03 | 13.37 | 19.09 | 14.08 | 16.33 | 13.53 |
| La | 36.47 | 43.91 | 86.78 | 45.23 | 24.6 | 15.66 | 20.77 | 29.57 |
| Ce | 82.7 | 93.35 | 203.5 | 97.23 | 51.67 | 32.76 | 46.84 | 61.55 |
| Pr | 9.06 | 10.75 | 23.5 | 11.06 | 5.83 | 3.69 | 5.5 | 6.92 |
| Nd | 36.29 | 43.35 | 96.28 | 44.5 | 23.17 | 15.1 | 23.38 | 27.24 |
| Sm | 6.71 | 8.17 | 18.09 | 8.34 | 4.43 | 2.59 | 4.56 | 5.23 |
| Eu | 1.22 | 1.59 | 4.09 | 1.64 | 0.95 | 0.5 | 1.14 | 1.36 |
| Gd | 6.6 | 8.42 | 16.13 | 8.36 | 4.58 | 2.56 | 4.66 | 5.7 |
| Tb | 0.81 | 1.08 | 1.7 | 1.06 | 0.59 | 0.28 | 0.57 | 0.77 |
| Dy | 4.12 | 5.63 | 6.99 | 5.45 | 3.3 | 1.67 | 3.24 | 4.46 |
| Y | 21.33 | 29.3 | 27.73 | 28.07 | 15.83 | 8.81 | 17.06 | 23.8 |
| Ho | 0.79 | 1.09 | 1.15 | 1.05 | 0.61 | 0.3 | 0.6 | 0.85 |
| Er | 2.21 | 3.06 | 2.94 | 2.93 | 1.8 | 0.94 | 1.71 | 2.43 |
| Tm | 0.31 | 0.43 | 0.39 | 0.41 | 0.24 | 0.11 | 0.21 | 0.31 |
| Yb | 2.03 | 2.79 | 2.5 | 2.68 | 1.69 | 0.89 | 1.44 | 2.08 |
| Lu | 0.3 | 0.42 | 0.35 | 0.4 | 0.23 | 0.11 | 0.2 | 0.29 |

- continued.

| Element | LY-1 | LY-2 | YC-1 | YC-2 | BL-1 | BL-2 | BL-C1-A | LNC-1 | LNC-2 | LH-1 |
| --- | --- | --- | --- | --- | --- | --- | --- | --- | --- | --- |
| Si | 94593 | 41160 | 60900 | 137667 | 71120 | 108033 | 94547 | 74013 | 114380 | 96367 |
| V | 178.79 | 135.88 | 97.97 | 66.18 | 78.22 | 38.34 | 110.48 | 68.32 | 85.14 | 78.91 |
| Cr | 34.01 | 42.58 | 54.05 | 13.3 | 17.68 | 15.77 | 31.89 | 32.42 | 22.09 | 94.74 |
| Co | 18.29 | 20.54 | 11.06 | 26.21 | 46.6 | 22.07 | 23.46 | 24.56 | 26.81 | 12.89 |
| Ni | 22.85 | 34.08 | 28.05 | 29.85 | 29.48 | 20.41 | 29.55 | 35.96 | 26.13 | 19.61 |
| As | 2.9 | 1.61 | 2.76 | 0.61 | 1.98 | 1.19 | 1.61 | 2.41 | 0.82 | 2.56 |
| Mo | 0.95 | 1.01 | 1.29 | 2.27 | 6.67 | 0.9 | 1.92 | 2.48 | 0.82 | 3.11 |
| Cd | 0.15 | 0.65 | 0.86 | 0.52 | 2.65 | 0.99 | 0.95 | 0.35 | 0.16 | 0.61 |
| W | 0.93 | 2.89 | 0.93 | 2.76 | 33.65 | 2.8 | 6.95 | 2.15 | 2.71 | 1.23 |
| Pb | 11.73 | 14.67 | 44.79 | 11.74 | 20.91 | 15.57 | 13.14 | 7.08 | 12.55 | 2.99 |
| La | 35.33 | 58.88 | 28.19 | 25.39 | 20.28 | 40.17 | 41.44 | 18.63 | 33.99 | 26.1 |
| Ce | 80.3 | 125.98 | 63.2 | 57.09 | 49 | 82.1 | 91.81 | 38.67 | 67.64 | 46.04 |
| Pr | 9.88 | 14.28 | 7.66 | 6.74 | 5.73 | 9.6 | 10.22 | 4.26 | 7.67 | 5.44 |
| Nd | 38.59 | 55.59 | 30.88 | 29.52 | 21.55 | 36.15 | 40.08 | 16.76 | 29.46 | 20.56 |
| Sm | 8.03 | 10.78 | 6.13 | 5.68 | 4.53 | 7.2 | 7.83 | 3.37 | 5.39 | 3.74 |
| Eu | 1.98 | 2.45 | 1.37 | 1.44 | 0.65 | 1.23 | 1.67 | 0.87 | 1.39 | 0.96 |
| Gd | 6.5 | 9.14 | 4.66 | 6.03 | 4.95 | 8.02 | 8.07 | 3.17 | 5.61 | 3.86 |
| Tb | 0.93 | 1.48 | 0.65 | 0.76 | 0.79 | 1.19 | 1.09 | 0.52 | 0.72 | 0.54 |
| Dy | 4.09 | 6.67 | 3.51 | 4.46 | 3.87 | 5.34 | 5 | 2.95 | 3.99 | 3.14 |
| Y | 20.3 | 31.7 | 23.09 | 25.15 | 25 | 30.38 | 24.76 | 16.48 | 20.03 | 16.56 |
| Ho | 0.74 | 1.28 | 0.77 | 0.84 | 0.84 | 1.11 | 0.97 | 0.57 | 0.74 | 0.59 |
| Er | 2.13 | 3.33 | 2.21 | 2.42 | 2.27 | 2.95 | 2.52 | 1.51 | 2.1 | 1.69 |
| Tm | 0.29 | 0.48 | 0.35 | 0.29 | 0.32 | 0.43 | 0.36 | 0.23 | 0.26 | 0.21 |
| Yb | 1.8 | 3.03 | 2.07 | 1.96 | 2.13 | 2.77 | 2.29 | 1.31 | 1.76 | 1.43 |
| Lu | 0.25 | 0.46 | 0.32 | 0.27 | 0.34 | 0.43 | 0.35 | 0.2 | 0.24 | 0.19 |

- continued.

| Element | LH-2 | XH-1 | XH-2 | HLS-1 | HLS-2 | BM-1 | BM-2 | BM-3 | HLW-1 | HLW-2 |
| --- | --- | --- | --- | --- | --- | --- | --- | --- | --- | --- |
| Si | 150733 | 52593 | 195067 | 74573 | 40413 | 194507 | 41767 | 126700 | 43400 | 64400 |
| V | 106.13 | 26.42 | 52.28 | 134.88 | 163.8 | 15.12 | 53.1 | 73.21 | 95.53 | 150.8 |
| Cr | 61.45 | 19.15 | 18.14 | 60.63 | 49.51 | 10.19 | 35.02 | 84.24 | 20.25 | 37.96 |
| Co | 27.58 | 18.76 | 24.12 | 16.4 | 22.57 | 8.45 | 18.25 | 15.51 | 12.55 | 19.14 |
| Ni | 39.1 | 30.87 | 26.84 | 24.84 | 32.9 | 13.78 | 23.72 | 18.81 | 17.85 | 25.82 |
| As | 1.92 | 2.4 | 0.34 | 2.59 | 7.9 | 2.56 | 2.51 | 2.69 | 4.17 | 1.42 |
| Mo | 0.78 | 5.78 | 0.44 | 4.11 | 4.22 | 4.02 | 1.57 | 2.63 | 2.27 | 1.19 |
| Cd | 0.25 | 2.01 | 1.1 | 0.09 | 0.45 | 0.42 | 0.91 | 0.34 | 0.77 | 0.48 |
| W | 2.64 | 0.84 | 4.84 | 0.68 | 1.38 | 1.14 | 2.15 | 2.94 | 0.54 | 1.05 |
| Pb | 13.63 | 6.08 | 16.86 | 24.62 | 14.42 | 8.22 | 3.26 | 18.05 | 9.75 | 11.28 |
| La | 28.55 | 5.07 | 6.68 | 24.93 | 35.45 | 10.39 | 17.08 | 15.07 | 24.21 | 35.54 |
| Ce | 53.65 | 10.04 | 15.33 | 51.43 | 69.48 | 17.04 | 34.84 | 25.28 | 51.06 | 70.96 |
| Pr | 6.21 | 1.23 | 1.8 | 6.25 | 8.49 | 2.46 | 4.21 | 2.81 | 6.42 | 8.26 |
| Nd | 22.85 | 4.94 | 6.94 | 27.15 | 34.48 | 12.21 | 16.8 | 10.26 | 25.38 | 33.34 |
| Sm | 4.78 | 1.51 | 1.61 | 6.33 | 7.34 | 2.84 | 3.35 | 2.14 | 5.04 | 6.5 |
| Eu | 1.33 | 0.27 | 0.21 | 1.85 | 1.93 | 0.83 | 0.91 | 0.66 | 1.21 | 1.66 |
| Gd | 4.65 | 1.64 | 1.72 | 6.04 | 7.32 | 3.26 | 3.07 | 2.21 | 5.36 | 6.57 |
| Tb | 0.73 | 0.36 | 0.29 | 0.86 | 1.14 | 0.55 | 0.49 | 0.4 | 0.76 | 0.91 |
| Dy | 3.78 | 2.1 | 1.46 | 4.74 | 5.38 | 2.58 | 2.64 | 2.43 | 4.28 | 4.76 |
| Y | 17.4 | 13.15 | 9.41 | 19.89 | 18.37 | 16.39 | 16.24 | 13.85 | 17.48 | 18.29 |
| Ho | 0.67 | 0.48 | 0.31 | 0.85 | 1.09 | 0.56 | 0.51 | 0.5 | 0.81 | 0.87 |
| Er | 1.82 | 1.37 | 0.87 | 2.1 | 3.06 | 1.68 | 1.49 | 1.34 | 2.26 | 2.4 |
| Tm | 0.23 | 0.2 | 0.13 | 0.28 | 0.4 | 0.2 | 0.19 | 0.19 | 0.3 | 0.32 |
| Yb | 1.58 | 1.22 | 0.87 | 1.56 | 2.61 | 1.08 | 1.33 | 1.17 | 2.07 | 2.08 |
| Lu | 0.22 | 0.18 | 0.14 | 0.22 | 0.36 | 0.15 | 0.19 | 0.16 | 0.29 | 0.28 |

- continued.

| Element | Average | China coal | UCC | CC_china_ |
| --- | --- | --- | --- | --- |
| Si | 108035 | 39527 | 310893 | 2.73 |
| V | 101.45 | 35.1 | 60 | 2.89 |
| Cr | 35.81 | 15.4 | 35 | 2.33 |
| Co | 23.29 | 7.08 | 10 | 3.29 |
| Ni | 31.48 | 13.7 | 20 | 2.30 |
| As | 3.31 | 3.79 | 1.5 | 0.87 |
| Mo | 2.06 | 3.08 | 1.5 | 0.67 |
| Cd | 0.86 | 0.25 | 0.1 | 3.45 |
| W | 3.31 | 1.08 | 2 | 3.07 |
| Pb | 15.13 | 15.1 | 20 | 1.00 |
| La | 30.17 | 22.5 | 30 | 1.34 |
| Ce | 64.17 | 46.7 | 64 | 1.37 |
| Pr | 7.50 | 6.42 | 7.1 | 1.17 |
| Nd | 29.83 | 22.3 | 26 | 1.34 |
| Sm | 5.90 | 4.07 | 4.5 | 1.45 |
| Eu | 1.30 | 0.84 | 0.88 | 1.54 |
| Gd | 5.74 | 4.65 | 3.8 | 1.23 |
| Tb | 0.81 | 0.62 | 0.64 | 1.31 |
| Dy | 4.22 | 3.74 | 3.5 | 1.13 |
| Y | 21.94 | 18.2 | 22 | 1.21 |
| Ho | 0.83 | 0.96 | 0.8 | 0.86 |
| Er | 2.31 | 1.79 | 2.3 | 1.29 |
| Tm | 0.32 | 0.64 | 0.33 | 0.50 |
| Yb | 2.08 | 2.08 | 2.2 | 1.00 |
| Lu | 0.30 | 0.38 | 0.32 | 0.79 |

**Table S4** Abundances of trace elements in Longtan Formation coal (mg/kg).

| Element | XHB1 | XHB2 | BLS1 | BLS2 |
| --- | --- | --- | --- | --- |
| Si | 74947 | 52593 | 58240 | 40460 |
| V | 162.72 | 92.86 | 100.47 | 84.42 |
| Cr | 47.24 | 20.07 | 21.61 | 16.71 |
| Co | 22.46 | 12.47 | 12.38 | 11.85 |
| Ni | 14.96 | 17.43 | 13.22 | 15.81 |
| As | 8.15 | 4.68 | 2.88 | 1.47 |
| Mo | 4.34 | 1.94 | 2.75 | 1.02 |
| Cd | 0.45 | 0.26 | 0.29 | 0.23 |
| Sb | 0.94 | 0.38 | 0.56 | 0.22 |
| Pb | 14.60 | 7.97 | 10.82 | 7.81 |
| La | 30.23 | 20.92 | 21.42 | 15.71 |
| Ce | 68.96 | 44.45 | 45.05 | 34.38 |
| Pr | 8.43 | 5.35 | 5.82 | 4.15 |
| Nd | 33.65 | 21.42 | 22.92 | 16.28 |
| Sm | 7.14 | 4.20 | 4.57 | 3.37 |
| Eu | 1.85 | 0.98 | 1.08 | 0.78 |
| Gd | 7.90 | 4.45 | 4.76 | 3.61 |
| Tb | 1.10 | 0.62 | 0.69 | 0.52 |
| Dy | 6.02 | 3.48 | 3.81 | 3.05 |
| Y | 20.86 | 18.49 | 14.38 | 16.71 |
| Ho | 1.05 | 0.65 | 0.73 | 0.56 |
| Er | 2.95 | 1.86 | 2.05 | 1.60 |
| Tm | 0.39 | 0.24 | 0.27 | 0.21 |
| Yb | 2.58 | 1.66 | 1.87 | 1.43 |
| Lu | 0.35 | 0.23 | 0.25 | 0.20 |

Table S5 Tests of Equality of Group Means.

|  | Wilks' Lambda | F | Df1 | Df2 | Sig. |
| --- | --- | --- | --- | --- | --- |
| V | 0.960 | 0.515 | 3 | 37 | 0.675 |
| Cr | 0.977 | 0.294 | 3 | 37 | 0.829 |
| Co | 0.746 | 4.209 | 3 | 37 | **0.012** |
| Ni | 0.576 | 9.093 | 3 | 37 | **0.000** |
| As | 0.799 | 3.094 | 3 | 37 | **0.039** |
| Mo | 0.945 | 0.712 | 3 | 37 | 0.551 |
| Cd | 0.780 | 3.475 | 3 | 37 | **0.026** |
| Sb | 0.949 | 0.659 | 3 | 37 | 0.582 |
| Pb | 0.943 | 0.743 | 3 | 37 | 0.534 |
| Si | 0.726 | 4.649 | 3 | 37 | **0.007** |

Table S6 Statistics of Wilks' Lambda value.

| Test of Function(s) | Wilks' Lambda | Chi-square | Df | Sig. |
| --- | --- | --- | --- | --- |
| 1 through3 | **0.320** | **40.460** | 15 | **0.000** |
| 2 through3 | 0.772 | 9.167 | 8 | 0.328 |
| 3 | 0.956 | 1.599 | 3 | 0.660 |

Table S7 Box's M test results of equality covariance matrixes

| Box's M | F | | | |
| --- | --- | --- | --- | --- |
|  | Approx. | Df1 | Df2 | Sig. |
| **114.604** | 2.621 | 30 | 835.273 | **0.000** |

Table S8 Eigenvalues of canonical discriminant function.

| Function | Eigenvalue | % of Variance | Cumulative % | Canonical Correlation |
| --- | --- | --- | --- | --- |
| 1 | 1.414^a^ | 83.3 | 83.3 | 0.765 |
| 2 | .238^a^ | 14.0 | 97.3 | 0.438 |
| 3 | .046^a^ | 2.7 | 100.0 | 0.210 |

Table S9 Structure Matrix.

| Function | Ni | Si | Cd | As | Co |
| --- | --- | --- | --- | --- | --- |
| 1 | .722^*^ | .515^*^ | .416^*^ | 0.107 | 0.471 |
| 2 | -0.010 | 0.087 | 0.384 | .993^*^ | -0.056 |
| 3 | 0.103 | 0.089 | -0.196 | -0.023 | .761^*^ |

Table S10 Canonical Discriminant Function Coefficients.

| Function | Co | Ni | As | Cd | Si | (常量) |
| --- | --- | --- | --- | --- | --- | --- |
| 1 | -0.821 | 6.705 | -0.774 | 2.443 | 3.596 | -15.938 |
| 2 | 0.396 | -0.974 | 4.350 | -0.132 | -0.420 | -0.627 |
| 3 | 9.851 | -4.862 | 1.103 | -3.756 | 0.404 | -6.726 |

Table S11 Zoning comparison of REY’ concentrations in coal (mg/kg).

|  | La | Ce | Pr | Nd | Sm | Eu | Gd | Tb |
| --- | --- | --- | --- | --- | --- | --- | --- | --- |
| Ⅰ | 22.07 | 48.21 | 5.94 | 23.57 | 4.82 | 1.17 | 5.18 | 0.73 |
| Ⅱ | 34.02 | 72.80 | 8.46 | 34.00 | 6.58 | 1.26 | 6.41 | 0.89 |
| Ⅲ | 27.12 | 57.08 | 6.75 | 26.70 | 5.44 | 1.14 | 5.26 | 0.80 |
| Ⅳ | **27.00** | **56.86** | **6.69** | **26.16** | **5.20** | **1.05** | **5.03** | **0.77** |
|  | Dy | Y | Ho | Er | Tm | Yb | Lu | REY |
| Ⅰ | 4.09 | 17.61 | 0.75 | 2.12 | 0.28 | 1.89 | 0.26 | 138.67 |
| Ⅱ | 4.65 | 24.01 | 0.90 | 2.54 | 0.37 | 2.36 | 0.36 | 199.59 |
| Ⅲ | 4.31 | 22.89 | 0.87 | 2.47 | 0.35 | 2.27 | 0.33 | 163.79 |
| Ⅳ | **4.25** | 23.99 | 0.87 | 2.51 | 0.36 | 2.35 | 0.34 | **163.43** |

Table S12 Zoning comparison of REY content and fractionation value in coal.

|  | LREY | HREY | LREY/HREY | (La/Yb)_N_ | (La/Sm)_N_ | (Gd/Yb)_N_ | δEu | δCe |
| --- | --- | --- | --- | --- | --- | --- | --- | --- |
| Ⅰ | 105.78 | 32.90 | 3.18 | 0.86 | 0.70 | 1.56 | 1.08 | 0.96 |
| Ⅱ | 157.11 | 42.49 | 3.68 | 1.06 | 0.77 | 1.58 | 0.91 | 0.98 |
| Ⅲ | 125.59 | 35.12 | 3.52 | 1.10 | 0.79 | 1.64 | 1.12 | 0.95 |
| Ⅳ | 125.21 | 40.10 | **3.00** | 0.89 | 0.74 | **1.38** | 1.00 | **0.95** |

$\delta\mathrm{Eu}_{N}={2Eu}_{N}/(\mathrm{Sm}_{N}+\mathrm{Gd}_{N})$,$\delta\mathrm{Ce}_{N}={2Ce}_{N}/(\mathrm{La}_{N}\times\Pr_{N})$

Subscript N indicates normalization with UCC abundance.

The description of Fisher discriminant method

Suppose there are k classifications (populations) F_1_, F_2,_ ⋯, F_k_ (i=1, 2, ⋯, k）, and the corresponding mean vector is μ^(1)^, μ^(2)^, ⋯, μ^(k)^, the corresponding covariance matrix is M^(1)^, M^(2)^, ⋯, M^(k)^. If a sample with the size of n_i_ is extracted from the total F_i_, that is:

$X_{a}^{i}=\left（ x_{a_{1}}^{i},x_{a_{2}}^{i},\cdots,x_{a_{p}}^{i} \right）^{T},（a=1, 2, \cdots, n; i=1, 2, \cdots, k）$ （1）

then:

$\mu^{T}X_{a}^{i}=\left（ \mu^{1}x_{a_{1}}^{i},\mu^{2}x_{a_{2}}^{i},\cdots,{\mu^{p}x}_{a_{p}}^{i} \right）^{T},(i=1, 2, \cdots, k)$ （2）

vector $\mu={(\mu_{1}, \mu_{2},\cdots,\mu_{p})}^{T}$ represents a direction in p-dimensional space; $Y={\mu_{1}}^{T}X$ is μ and the inner product of X, that is, X is the μ projection on the axis.

The projection of sample X_i_ on the spatial axis can be expressed as:

$$\left\{ \begin{aligned} \bar{X}^{i}=\frac{1}{n_{i}}\sum_{i=1}^{n_{i}} X_{a}^{i} \\ \bar{X}=\frac{1}{n}\sum_{i=1}^{k} \sum_{a=1}^{n_{i}} X_{a}^{i}, k=\sum_{i=1}^{k} n_{i} \end{aligned} (3) \right.$$

$\bar{X}^{i}$ is the average value of the selected samples, and $\bar{X}$is the average of the total samples. In this case, the intragroup deviation e of a group of samples is:

$$e=\sum_{i=1}^{k} \sum_{a=1}^{n_{i}} {(\mu^{T}X_{a}^{i}-\mu^{T}\bar{X}^{i})}^{2}=\mu^{T}\left\{ \sum_{i=1}^{k} G_{i} \right\}\mu=\mu^{T}W\mu, \left( W=\left\{ \sum_{i=1}^{k} G_{i} \right\} \right) (4)$$

where $G_{i}$ is the sample $T_{i}$ on the spatial axis (sample size =$n_{i}$) projection, with a sample difference of $X_{a}^{i}$. W is the "total dispersion within class" matrix. The deviation b between sample groups is:

$$\left\{ \begin{aligned} b=\mu^{T}\left[ n_{i}\sum_{i=1}^{k} {(\bar{X}^{i}-\bar{X})(\bar{X}^{i}-\bar{X})}^{T} \right]\mu=\mu^{T}B\mu\\ B=\left[ n_{i}\sum_{i=1}^{k} {(\bar{X}^{i}-\bar{X})(\bar{X}^{i}-\bar{X})}^{T} \right] \end{aligned} (5) \right.$$

where B is the "within class dispersion" matrix of the sample. When using the total discrimination function, $\Phi_{p}$ can be expressed as:

$\Phi_{p}=\frac{b}{e}=\frac{\mu^{T}B\mu}{\mu^{T}W\mu}$ （6）

To obtain a $\Phi_{p}$ maxima, the solution must be unique and needs to meet the $\mu^{T}W\mu$=1 condition. Thus, the problem was transformed into the condition $\mu^{T}W\mu$=1. Lower request $\mu^{T}B\mu$ Ratio to maximum μ。

If $FA=\mu^{T}B\mu-\lambda(\mu^{T}W\mu-1)$, its partial differential is calculated and is set equal to 0 to obtain:

$\frac{\partial FA}{\partial\mu}=2B\mu-2\lambda W\mu=0$ （7）

In Equation (7), λ is its eigenvalue. Through simplification, the following equation is obtained:

${(W}^{-1}B-\lambda)\mu=0$ （8）

The maximum eigenvalue $W^{-1}B$ and eigenvector μ can be obtained from the above formula. Thus, the discriminant function is obtained.

Table S13 ASMR of lung cancer zoning table.

| Zoning | Township |
| --- | --- |
| Normal | Wude, Reshui, Huangnihe, Gugan |
| Ⅰ-(Low) | Xize, Yangliu, Shuanghe, Adu, Puli, Fucun, Shibalianshan |
| Ⅱ-(Medium) | Banqiao, Tangtang, Tianba, Geyi, Luoshui, Delu, Lefeng, Wenxing, Laochang |
| Ⅲ-(High) | Longtan, Xining, Wanshui, Hongqiao, Baoshan, Dongshan,  Yangchang, Haidai, Zhongan, Mohong, Yingshang, Zhuyuan |
| Ⅳ-(Ultrahigh) | Laibing, Longchang, Shuanglong, Houshuo, Dahe |

Table S14 Coal sample information (Chen et al., 2023).

| Stratigraphic units | Zoning | Coal name | Layer | Sources |
| --- | --- | --- | --- | --- |
| Longtan Formation | Ⅰ-(Low) | Xiaohebian coal | C10 | This study |
|  |  | Bailongshan coal | C2 | This study |
| Xuanwei Formation | Ⅱ-(Medium) | Xinde coal | C2、C3 | Dai et al., 2014 |
|  |  | Xionggou coal | - | This study |
|  |  | Jiubao coal | K2、K5 | This study |
|  | Ⅲ-(High) | Laoying coal | K3 | This study |
|  |  | Yongchang coal | K15、K16 | This study |
|  |  | Lianying coal | - | Zheng, 2018 |
|  |  | Bole coal | C1、C2 | Li, 2015; This study |
|  |  | Longhai coal | K8 | This study |
|  |  | Bumu coal | M11 | This study |
|  |  | Helewu coal | C1 | This study |
|  | Ⅳ-(Ultrahigh) | Yantang coal | C1 | Dai et al., 2008; This study |
|  |  | Daqingpo coal | Y1-2、Y4 | This study |
|  |  | Laoniuchang coal | - | This study |
|  |  | Xinhua Coal | C7 | This study |
|  |  | Heilushan coal | M4+1 | This study |

**Reference:**

Chen, Z., Shi, Z., Ni, S., Hu, J. 2023. Environmental geochemical characteristics of Xuanwei Formation coal and their controlling geological factors, with comments on the relationship with lung cancer incidence and distribution. Geological Society of London.

<https://doi.org/10.6084/m9.figshare.c.6444476.v1>.

Table S15 Zoning comparison of the carcinogenic element concentrations in the coal samples (mg/kg).

| Zoning | Si | V | Cr | Co | Ni | As | Mo | Cd | Sb | Pb |
| --- | --- | --- | --- | --- | --- | --- | --- | --- | --- | --- |
| Ⅰ | 56560 | 110.12 | 26.41 | 14.79 | 15.36 | 4.30 | 2.51 | 0.31 | 0.53 | 10.30 |
| Ⅱ | 119093 | 129.96 | 34.21 | 24.60 | 29.86 | 2.07 | 1.98 | 0.57 | 0.74 | 14.94 |
| Ⅲ | 100621 | 86.35 | 34.95 | 21.18 | 26.65 | 1.95 | 1.85 | 0.63 | 0.74 | 14.60 |
| Ⅳ | 113195 | 113.13 | **37.20** | **25.63** | **38.35** | 5.76 | 2.40 | **1.30** | 0.42 | **15.87** |

Table S16 Varimax rotated component matrix loading for carcinogenic elements

| \| **Rotated Component Matrix** \| \| \| \| \| \| \| --- \| --- \| --- \| --- \| --- \| --- \| \| Element \| Component \| \| \| \| \| \| PC1 \| PC2 \| PC3 \| PC4 \| PC5 \| \| Co \| **0.893** \| - \| - \| 0.151 \| 0.151 \| \| Ni \| **0.840** \| 0.106 \| 0.261 \| -0.146 \| 0.199 \| \| As \| **-** \| **0.850** \| 0.280 \| -0.129 \| - \| \| Cd \| 0.491 \| **0.699** \| -0.255 \| 0.187 \| - \| \| Mo \| -0.254 \| **0.626** \| 0.181 \| 0.595 \| 0.117 \| \| Cr \| - \| 0.123 \| **0.862** \| - \| 0.113 \| \| V \| 0.336 \| - \| **0.847** \| - \| -0.130 \| \| Sb \| 0.109 \| - \| **-** \| **0.958** \| - \| \| Si \| - \| 0.340 \| -0.109 \| 0.144 \| **0.813** \| \| Pb \| 0.324 \| -0.206 \| 0.125 \| - \| **0.728** \| \| Eigen value \| 2.744 \| 1.866 \| 1.652 \| 1.051 \| 0.950 \| \| % of variance \| 27.444 \| 18.664 \| 16.521 \| 10.507 \| 9.496 \| \| Cumulative % \| 27.444 \| 46.108 \| 62.629 \| 73.137 \| 82.633 \| |
| --- | --- | --- | --- | --- | --- | --- | --- | --- | --- | --- | --- | --- | --- | --- | --- | --- | --- | --- | --- | --- | --- | --- | --- | --- | --- | --- | --- | --- | --- | --- | --- | --- | --- | --- | --- | --- | --- | --- | --- | --- | --- | --- | --- | --- | --- | --- | --- | --- | --- | --- | --- | --- | --- | --- | --- | --- | --- | --- | --- | --- | --- | --- | --- | --- | --- | --- | --- | --- | --- | --- | --- | --- | --- | --- | --- | --- | --- | --- | --- | --- | --- | --- | --- | --- | --- | --- | --- | --- | --- | --- | --- | --- | --- | --- | --- |

Extraction Method: Principal component analysis.

Rotation Method: Varimax with Kaiser normalization.

The bold values indicate loading coefficients > 0.6.


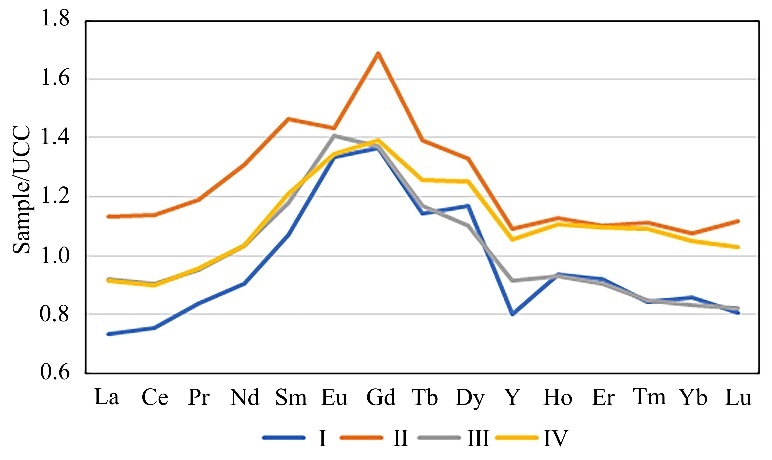


Fig. S1. The distribution pattern of REY in coal (UCC normalization).

Table S17 Toxicological characteristics of carcinogenic elements.

| Elements | Classification (IARC, 2019) | Symptom | Ref |
| --- | --- | --- | --- |
| Silicon (Si) | 1 (crystalline silica), 3 (amorphous silica) | Pulmonary fibrosis, lung cancer | (Napierska et al., 2010; Croissant et al., 2020) |
| Vanadium (V) | 2B (V_2_O_5_) | Rhinitis, pharyngitis, cough, tracheobronchitis, and bronchopneumonia. | (Ghosh et al., 2014) |
| Chromium (Cr) | 1 (Cr^6+^), 3(Cr^3+^) | Lung cancer. | (Proctor et al., 2014;  Rager et al., 2019) |
| Cobalt (Co) | 2A (Cobalt Compounds) | Lung cancer, asthma and alveolitis. | (Smith et al., 2014) |
| Nickel (Ni) | 1 (Nickel compounds) | Lung cancer. | (Grimsrud et al., 2002; Pesch et al., 2019;  Guo et al., 2021) |
| Arsenic (As) | 1 (Arsenic and inorganic  arsenic compounds) | Skin (keratoses, melanosis, skin tumors),  perforated nasal septum, and visceral cancers. | (Hu et al., 2021;  Lamm et al., 2021) |
| Molybdenum (Mo) | 2B (Mo_2_O_3_) | Anemia, reduced red blood cell counts, and joint deformities. | (Božinović et al., 2020;  Reutova et al., 2022) |
| Cadmium (Cd) | 1 (Cadmium and  cadmium compounds) | Lung cancer, kidney damage, osteoporosis. | (Person et al., 2013;  Wang et al., 2019) |
| Antimony (Sb) | 2B (Sb_2_O_3_) | Pneumonitis, hemolysis, vomiting, joint and muscle pains. | (Boreiko and Rossman, 2020; Nishad and  Bhaskarapillai, 2021) |
| Lead (Pb) | 2A (Lead compounds  (inorganic)) | Nervous system. | (Boskabady et al., 2018;  Huang et al., 2021) |

**Reference:**

Boskabady, M., Marefati, N., Farkhondeh, T., Shakeri, F., Farshbaf, A., Boskabady, M., 2018. The effect of environmental lead exposure on human health and the contribution of inflammatory mechanisms, a review. Environ. Int., 120, 404-420. <https://doi.org/10.1016/j.envint.2018.08.013>.

Božinović, K., Nestić, D., Centa, U. G., et al., 2020. In-vitro toxicity of molybdenum trioxide nanoparticles on human keratinocytes. Toxicol., 152564.

<https://doi.org/10.1016/j.tox.2020.152564>.

Boreiko, C. J., Rossman, T. G. 2020. Antimony and its compounds: Health impacts related to pulmonary toxicity, cancer, and genotoxicity. Toxicol. Appl. Pharmacol., 115156.

<https://doi.org/10.1016/j.taap.2020.115156>.

Croissant, J. G., Butler, K. S., Zink, J. I., Brinker, C. J. 2020. Synthetic amorphous silica nanoparticles: toxicity, biomedical and environmental implications. Nat. Rev Mater., 5(12), 886-909.

<https://doi.org/10.1038/s41578-020-0230-0>.

Grimsrud, T. K., Berge, S. R., Haldorsen, T., Andersen, A. 2002. Exposure to different forms of nickel and risk of lung cancer. Am. J epidemiol., 156(12), 1123-1132.

<https://doi.org/10.1093/aje/kwf165>.

Ghosh, S.K., Saha, R. Saha, B. 2014. Toxicity of inorganic vanadium compounds. Res. Chem. Intermed., 41, 4873-4897. <https://doi.org/10.1007/s11164-014-1573-1>.

Guo, H., Deng, H., Liu, H. et al. 2021. Nickel carcinogenesis mechanism: cell cycle dysregulation. Environ. Sci. Pollut. Res., 28, 4893-4901. <https://doi.org/10.1007/s11356-020-11764-2>.

Hu, Y., Xiao, T., Zhang, A. 2021. Associations between and risks of trace elements related to skin and liver damage induced by arsenic from coal burning. Ecotoxi. Environ. Safe., 208, 111719.

<https://doi.org/10.1016/j.ecoenv.2020.111719>.

Huang. H., Jin. Y., Chen. C., et al., 2021. A toxicity pathway-based approach for modeling the mode of action framework of lead-induced neurotoxicity. Environ. Res., 199: 111328.

<https://doi.org/10.1016/j.envres.2021.111328>.

Lamm, S. H., Boroje, I. J., Ferdosi, H., Ahn, J. 2021. A review of low-dose arsenic risks and human cancers. Toxicol., 456, 152768. <https://doi.org/10.1016/j.tox.2021.152768>.

Napierska, D., Thomassen, L. C., Lison, D., Martens, J. A., Hoet, P. H. 2010. The nanosilica hazard: another variable entity. Part. Fibre Toxicol., 7(1), 1-32. <https://doi.org/10.1186/1743-8977-7-39>.

Nishad, P. A., Bhaskarapillai, A. 2021. Antimony, a pollutant of emerging concern: A review on industrial sources and remediation technologies. Chemosphere, 277, 130252.

<https://doi.org/10.1016/j.chemosphere.2021.130>.

Person, R. J., Tokar, E. J., Xu, Y., Orihuela, R., Ngalame, N. N. O., Waalkes, M. P. 2013. Chronic cadmium exposure in vitro induces cancer cell characteristics in human lung cells. Toxicol. Appl. Pharmacol., 273(2), 281-288. <https://doi.org/10.1016/j.taap.2013.06.013>.

Proctor, D. M., Suh, M., Campleman, S. L., Thompson, C. M. 2014. Assessment of the mode of action for hexavalent chromium-induced lung cancer following inhalation exposures. Toxicol., 325, 160-179. <https://doi.org/10.1016/j.tox.2014.08.009>.

Pesch, B., Kendzia, B., Pohlabeln, H., et al., 2019. Exposure to Welding Fumes, Hexavalent Chromium, or Nickel and Lung Cancer Risk. Am. J. Epidemiol., 188:1984-1993.

<https://doi.org/10.1093/aje/kwz187>.

Rager, J. E., Suh, M., Chappell, G. A., Thompson, C. M., Proctor, D. M. 2019. Review of transcriptomic responses to hexavalent chromium exposure in lung cells supports a role of epigenetic mediators in carcinogenesis. Toxicol. Lett., 305, 40-50. <https://doi.org/10.1016/j.toxlet.2019.01.011>.

Reutova, N.V., Reutova, T.V., Dreeva, F.R. et al. 2022. Long-term impact of the Tyrnyauz tungsten–molybdenum mining and processing factory waste on environmental pollution and children's population. Environ. Geochem. Health., <https://doi.org/10.1007/s10653-022-01221-z>.

Smith, L. J., Holmes, A. L., Kandpal, S. K., Mason, M. D., Zheng, T., Wise, J. P. 2014. The cytotoxicity and genotoxicity of soluble and particulate cobalt in human lung fibroblast cells. Toxicol. Appl. Pharmacol., 278(3), 259-265. <https://doi.org/10.1016/j.taap.2014.05.002>.

Wang, Y., Shi, L., Li, J., Wang, H., Yang, H. 2019. The roles of TG-interacting factor in cadmium exposure-promoted invasion and migration of lung cancer cells. Toxicol. in Vitro., 61, 104630.

<https://doi.org/10.1016/j.tiv.2019.104630>.

Table S18 data.heatmap.txt

|  | Si | V | Cr | Co | Ni | As | Mo | Cd | Sb | Pb | La | Ce | Pr | Nd | Sm | Eu | Gd | Tb | Dy | Y | Ho | Er | Tm | Yb | Lu |
| --- | --- | --- | --- | --- | --- | --- | --- | --- | --- | --- | --- | --- | --- | --- | --- | --- | --- | --- | --- | --- | --- | --- | --- | --- | --- |
| YT-1 | 2.20 | 2.03 | 1.64 | 1.46 | 1.85 | 0.62 | 0.55 | 0.42 | 0.03 | 1.50 | 1.40 | 1.72 | 0.84 | 1.37 | 0.74 | 0.21 | 0.70 | 0.26 | 0.80 | 1.48 | 0.34 | 0.68 | 0.21 | 0.74 | 0.20 |
| YT-2 | 2.23 | 2.28 | 1.65 | 1.40 | 1.63 | 0.95 | 0.54 | 0.53 | 0.12 | 0.92 | 1.60 | 1.96 | 1.03 | 1.58 | 0.88 | 0.34 | 0.89 | 0.29 | 0.78 | 1.44 | 0.31 | 0.58 | 0.14 | 0.54 | 0.14 |
| YT-B1 | 2.11 | 2.03 | 1.26 | 1.51 | 1.62 | 1.49 | 0.57 | 0.59 | 0.17 | 1.30 | 1.43 | 1.76 | 0.91 | 1.45 | 0.83 | 0.30 | 0.79 | 0.27 | 0.78 | 1.49 | 0.30 | 0.60 | 0.16 | 0.58 | 0.15 |
| YT-B2 | 2.15 | 2.22 | 1.57 | 1.46 | 1.61 | 0.91 | 0.43 | 0.54 | 0.18 | 1.23 | 1.46 | 1.72 | 0.88 | 1.41 | 0.75 | 0.34 | 0.71 | 0.21 | 0.64 | 1.30 | 0.22 | 0.46 | 0.11 | 0.45 | 0.10 |
| YT-B3 | 1.93 | 1.45 | 1.00 | 1.40 | 1.45 | 0.59 | 0.26 | 0.38 | 0.13 | 1.28 | 1.28 | 1.59 | 0.79 | 1.32 | 0.75 | 0.26 | 0.76 | 0.25 | 0.72 | 1.38 | 0.26 | 0.51 | 0.12 | 0.48 | 0.11 |
| DQP-Y1-2 | 2.07 | 2.18 | 1.94 | 1.48 | 1.60 | 0.61 | 0.44 | 0.33 | 0.05 | 1.21 | 1.65 | 1.95 | 1.08 | 1.67 | 0.98 | 0.51 | 0.95 | 0.33 | 0.85 | 1.59 | 0.35 | 0.64 | 0.17 | 0.56 | 0.14 |
| DQP-Y4 | 2.11 | 2.29 | 1.68 | 1.57 | 1.81 | 0.72 | 0.38 | 0.23 | 0.29 | 1.29 | 1.73 | 2.09 | 1.15 | 1.73 | 1.08 | 0.45 | 1.06 | 0.38 | 0.90 | 1.60 | 0.40 | 0.75 | 0.22 | 0.74 | 0.21 |
| LNC-1 | 2.15 | 1.84 | 1.52 | 1.41 | 1.57 | 0.53 | 0.54 | 0.13 | 0.26 | 0.91 | 1.29 | 1.60 | 0.72 | 1.25 | 0.64 | 0.27 | 0.62 | 0.18 | 0.60 | 1.24 | 0.20 | 0.40 | 0.09 | 0.36 | 0.08 |
| LNC-2 | 2.02 | 1.94 | 1.36 | 1.44 | 1.43 | 0.26 | 0.26 | 0.06 | 0.07 | 1.13 | 1.54 | 1.84 | 0.94 | 1.48 | 0.81 | 0.38 | 0.82 | 0.24 | 0.70 | 1.32 | 0.24 | 0.49 | 0.10 | 0.44 | 0.09 |
| XH-1 | 2.10 | 1.44 | 1.30 | 1.30 | 1.50 | 0.53 | 0.83 | 0.48 | 0.13 | 0.85 | 0.78 | 1.04 | 0.35 | 0.77 | 0.40 | 0.10 | 0.42 | 0.13 | 0.49 | 1.15 | 0.17 | 0.37 | 0.08 | 0.35 | 0.07 |
| XH-2 | 1.94 | 1.73 | 1.28 | 1.40 | 1.44 | 0.13 | 0.16 | 0.32 | 0.06 | 1.25 | 0.89 | 1.21 | 0.45 | 0.90 | 0.42 | 0.08 | 0.43 | 0.11 | 0.39 | 1.02 | 0.12 | 0.27 | 0.05 | 0.27 | 0.06 |
| HLS-1 | 2.15 | 2.13 | 1.79 | 1.24 | 1.41 | 0.56 | 0.71 | 0.04 | 0.26 | 1.41 | 1.41 | 1.72 | 0.86 | 1.45 | 0.87 | 0.45 | 0.85 | 0.27 | 0.76 | 1.32 | 0.27 | 0.49 | 0.11 | 0.41 | 0.09 |
| HLS-2 | 2.08 | 2.22 | 1.70 | 1.37 | 1.53 | 0.95 | 0.72 | 0.16 | 0.12 | 1.19 | 1.56 | 1.85 | 0.98 | 1.55 | 0.92 | 0.47 | 0.92 | 0.33 | 0.80 | 1.29 | 0.32 | 0.61 | 0.15 | 0.56 | 0.13 |
| JB-K5 | 2.15 | 1.52 | 1.52 | 1.32 | 1.30 | 0.55 | 0.51 | 0.16 | 0.16 | 1.29 | 1.46 | 1.78 | 0.91 | 1.46 | 0.83 | 0.28 | 0.79 | 0.24 | 0.67 | 1.30 | 0.23 | 0.48 | 0.11 | 0.45 | 0.10 |
| JB-K2 | 2.07 | 1.57 | 1.23 | 1.35 | 1.31 | 0.25 | 0.34 | 0.24 | 0.33 | 1.20 | 1.36 | 1.71 | 0.84 | 1.39 | 0.78 | 0.28 | 0.79 | 0.25 | 0.70 | 1.37 | 0.26 | 0.53 | 0.13 | 0.52 | 0.13 |
| XG-1 | 2.03 | 1.62 | 1.45 | 1.43 | 1.48 | 0.61 | 0.37 | 0.21 | 0.05 | 1.17 | 1.57 | 1.92 | 1.00 | 1.57 | 0.89 | 0.35 | 0.88 | 0.26 | 0.71 | 1.35 | 0.25 | 0.51 | 0.12 | 0.48 | 0.11 |
| XG-2 | 2.09 | 1.56 | 1.70 | 1.41 | 1.46 | 0.43 | 0.50 | 0.29 | 0.25 | 1.15 | 1.65 | 1.97 | 1.07 | 1.65 | 0.96 | 0.41 | 0.97 | 0.32 | 0.82 | 1.48 | 0.32 | 0.61 | 0.16 | 0.58 | 0.15 |
| XD-C-2 | 1.98 | 2.41 | 1.72 | 1.45 | 1.60 | 0.63 | 0.54 | 0.16 | 0.17 | 1.26 | 1.94 | 2.31 | 1.39 | 1.99 | 1.28 | 0.71 | 1.23 | 0.43 | 0.90 | 1.46 | 0.33 | 0.60 | 0.14 | 0.54 | 0.13 |
| XD-C-3 | 1.62 | 2.52 | 1.57 | 1.47 | 1.73 | 0.32 | 0.50 | 0.18 | 0.33 | 1.16 | 1.66 | 1.99 | 1.08 | 1.66 | 0.97 | 0.42 | 0.97 | 0.31 | 0.81 | 1.46 | 0.31 | 0.59 | 0.15 | 0.57 | 0.15 |
| LY-1234-A | 1.79 | 1.88 | 1.23 | 1.37 | 1.53 | 0.42 | 0.22 | 0.09 | 0.09 | 1.30 | 1.41 | 1.72 | 0.83 | 1.38 | 0.73 | 0.29 | 0.75 | 0.20 | 0.63 | 1.23 | 0.21 | 0.45 | 0.09 | 0.43 | 0.09 |
| LY-678-A | 2.14 | 1.71 | 1.14 | 1.38 | 1.49 | 0.18 | 0.10 | 0.02 | 0.03 | 1.18 | 1.22 | 1.53 | 0.67 | 1.21 | 0.56 | 0.18 | 0.55 | 0.11 | 0.43 | 0.99 | 0.11 | 0.29 | 0.05 | 0.28 | 0.05 |
| LY-91011-A | 1.86 | 1.81 | 1.22 | 1.45 | 1.51 | 0.34 | 0.32 | 0.13 | 0.07 | 1.24 | 1.34 | 1.68 | 0.81 | 1.39 | 0.75 | 0.33 | 0.75 | 0.20 | 0.63 | 1.26 | 0.20 | 0.43 | 0.08 | 0.39 | 0.08 |
| LY-12131415-A | 2.04 | 1.94 | 1.51 | 1.43 | 1.52 | 0.34 | 0.23 | 0.08 | 0.07 | 1.16 | 1.49 | 1.80 | 0.90 | 1.45 | 0.79 | 0.37 | 0.83 | 0.25 | 0.74 | 1.39 | 0.27 | 0.54 | 0.12 | 0.49 | 0.11 |
| LY-1 | 1.98 | 2.25 | 1.54 | 1.29 | 1.38 | 0.59 | 0.29 | 0.06 | 0.03 | 1.10 | 1.56 | 1.91 | 1.04 | 1.60 | 0.96 | 0.47 | 0.88 | 0.29 | 0.71 | 1.33 | 0.24 | 0.50 | 0.11 | 0.45 | 0.10 |
| LY-2 | 1.88 | 2.14 | 1.64 | 1.33 | 1.55 | 0.42 | 0.30 | 0.22 | 0.32 | 1.20 | 1.78 | 2.10 | 1.18 | 1.75 | 1.07 | 0.54 | 1.01 | 0.39 | 0.88 | 1.51 | 0.36 | 0.64 | 0.17 | 0.61 | 0.16 |
| YC-1 | 2.06 | 2.00 | 1.74 | 1.08 | 1.46 | 0.58 | 0.36 | 0.27 | 0.16 | 1.66 | 1.47 | 1.81 | 0.94 | 1.50 | 0.85 | 0.37 | 0.75 | 0.22 | 0.65 | 1.38 | 0.25 | 0.51 | 0.13 | 0.49 | 0.12 |
| YC-2 | 1.99 | 1.83 | 1.16 | 1.43 | 1.49 | 0.21 | 0.51 | 0.18 | 0.37 | 1.11 | 1.42 | 1.76 | 0.89 | 1.48 | 0.82 | 0.39 | 0.85 | 0.25 | 0.74 | 1.42 | 0.26 | 0.53 | 0.11 | 0.47 | 0.10 |
| BL-1 | 2.18 | 1.90 | 1.27 | 1.68 | 1.48 | 0.47 | 0.88 | 0.56 | 0.64 | 1.34 | 1.33 | 1.70 | 0.83 | 1.35 | 0.74 | 0.22 | 0.77 | 0.25 | 0.69 | 1.41 | 0.26 | 0.51 | 0.12 | 0.50 | 0.13 |
| BL-2 | 1.73 | 1.59 | 1.22 | 1.36 | 1.33 | 0.34 | 0.28 | 0.30 | 0.43 | 1.22 | 1.61 | 1.92 | 1.03 | 1.57 | 0.91 | 0.35 | 0.96 | 0.34 | 0.80 | 1.50 | 0.32 | 0.60 | 0.16 | 0.58 | 0.16 |
| BL-C1-A | 2.29 | 2.05 | 1.52 | 1.39 | 1.49 | 0.42 | 0.47 | 0.29 | 0.38 | 1.15 | 1.63 | 1.97 | 1.05 | 1.61 | 0.95 | 0.43 | 0.96 | 0.32 | 0.78 | 1.41 | 0.29 | 0.55 | 0.13 | 0.52 | 0.13 |
| LH-1 | 1.88 | 1.90 | 1.98 | 1.14 | 1.31 | 0.55 | 0.61 | 0.21 | 0.19 | 0.60 | 1.43 | 1.67 | 0.81 | 1.33 | 0.68 | 0.29 | 0.69 | 0.19 | 0.62 | 1.24 | 0.20 | 0.43 | 0.08 | 0.39 | 0.08 |
| LH-2 | 1.62 | 2.03 | 1.80 | 1.46 | 1.60 | 0.47 | 0.25 | 0.10 | 0.05 | 1.17 | 1.47 | 1.74 | 0.86 | 1.38 | 0.76 | 0.37 | 0.75 | 0.24 | 0.68 | 1.26 | 0.22 | 0.45 | 0.09 | 0.41 | 0.09 |
| BM-1 | 2.29 | 1.21 | 1.05 | 0.98 | 1.17 | 0.55 | 0.70 | 0.15 | 0.26 | 0.96 | 1.06 | 1.26 | 0.54 | 1.12 | 0.58 | 0.26 | 0.63 | 0.19 | 0.55 | 1.24 | 0.19 | 0.43 | 0.08 | 0.32 | 0.06 |
| BM-2 | 1.63 | 1.73 | 1.56 | 1.28 | 1.39 | 0.55 | 0.41 | 0.28 | 0.15 | 0.63 | 1.26 | 1.55 | 0.72 | 1.25 | 0.64 | 0.28 | 0.61 | 0.17 | 0.56 | 1.24 | 0.18 | 0.40 | 0.08 | 0.37 | 0.08 |
| BM-3 | 2.11 | 1.87 | 1.93 | 1.22 | 1.30 | 0.57 | 0.56 | 0.13 | 0.17 | 1.28 | 1.21 | 1.42 | 0.58 | 1.05 | 0.50 | 0.22 | 0.51 | 0.15 | 0.54 | 1.17 | 0.18 | 0.37 | 0.08 | 0.34 | 0.06 |
| HLW-1 | 1.65 | 1.98 | 1.33 | 1.13 | 1.28 | 0.71 | 0.51 | 0.25 | 0.08 | 1.03 | 1.40 | 1.72 | 0.87 | 1.42 | 0.78 | 0.34 | 0.80 | 0.25 | 0.72 | 1.27 | 0.26 | 0.51 | 0.11 | 0.49 | 0.11 |
| HLW-2 | 1.82 | 2.18 | 1.59 | 1.30 | 1.43 | 0.38 | 0.34 | 0.17 | 0.20 | 1.09 | 1.56 | 1.86 | 0.97 | 1.54 | 0.88 | 0.42 | 0.88 | 0.28 | 0.76 | 1.29 | 0.27 | 0.53 | 0.12 | 0.49 | 0.11 |
| XHB-1 | 1.88 | 2.21 | 1.68 | 1.37 | 1.20 | 0.96 | 0.73 | 0.16 | 0.29 | 1.19 | 1.49 | 1.84 | 0.97 | 1.54 | 0.91 | 0.45 | 0.95 | 0.32 | 0.85 | 1.34 | 0.31 | 0.60 | 0.14 | 0.55 | 0.13 |
| XHB-2 | 1.73 | 1.97 | 1.32 | 1.13 | 1.27 | 0.75 | 0.47 | 0.10 | 0.14 | 0.95 | 1.34 | 1.66 | 0.80 | 1.35 | 0.72 | 0.30 | 0.74 | 0.21 | 0.65 | 1.29 | 0.22 | 0.46 | 0.09 | 0.42 | 0.09 |
| BLS-1 | 1.77 | 2.01 | 1.35 | 1.13 | 1.15 | 0.59 | 0.57 | 0.11 | 0.19 | 1.07 | 1.35 | 1.66 | 0.83 | 1.38 | 0.75 | 0.32 | 0.76 | 0.23 | 0.68 | 1.19 | 0.24 | 0.48 | 0.10 | 0.46 | 0.10 |
| BLS-2 | 1.62 | 1.93 | 1.25 | 1.11 | 1.23 | 0.39 | 0.31 | 0.09 | 0.09 | 0.94 | 1.22 | 1.55 | 0.71 | 1.24 | 0.64 | 0.25 | 0.66 | 0.18 | 0.61 | 1.25 | 0.19 | 0.41 | 0.08 | 0.39 | 0.08 |

Table S19 sample.class.txt

|  | Group1 |
| --- | --- |
| Si | 1 |
| V | 2B |
| Cr | 1 |
| Co | 2B |
| Ni | 1 |
| As | 1 |
| Mo | 2B |
| Cd | 1 |
| Sb | 2B |
| Pb | 2A |
| La | REY |
| Ce | REY |
| Pr | REY |
| Nd | REY |
| Sm | REY |
| Eu | REY |
| Gd | REY |
| Tb | REY |
| Dy | REY |
| Y | REY |
| Ho | REY |
| Er | REY |
| Tm | REY |
| Yb | REY |
| Lu | REY |

Table S20 gene.class.txt

|  | Group2 |
| --- | --- |
| YT-1 | Ⅳ |
| YT-2 | Ⅳ |
| YT-B1 | Ⅳ |
| YT-B2 | Ⅳ |
| YT-B3 | Ⅳ |
| DQP-Y1-2 | Ⅳ |
| DQP-Y4 | Ⅳ |
| LNC-1 | Ⅳ |
| LNC-2 | Ⅳ |
| XH-1 | Ⅳ |
| XH-2 | Ⅳ |
| HLS-1 | Ⅳ |
| HLS-2 | Ⅳ |
| JB-K5 | Ⅱ |
| JB-K2 | Ⅱ |
| XG-1 | Ⅱ |
| XG-2 | Ⅱ |
| XD-C-2 | Ⅱ |
| XD-C-3 | Ⅱ |
| LY-1234-A | Ⅲ |
| LY-678-A | Ⅲ |
| LY-91011-A | Ⅲ |
| LY-12131415-A | Ⅲ |
| LY-1 | Ⅲ |
| LY-2 | Ⅲ |
| YC-1 | Ⅲ |
| YC-2 | Ⅲ |
| BL-1 | Ⅲ |
| BL-2 | Ⅲ |
| BL-C1-A | Ⅲ |
| LH-1 | Ⅲ |
| LH-2 | Ⅲ |
| BM-1 | Ⅲ |
| BM-2 | Ⅲ |
| BM-3 | Ⅲ |
| HLW-1 | Ⅲ |
| HLW-2 | Ⅲ |
| XHB-1 | Ⅰ |
| XHB-2 | Ⅰ |
| BLS-1 | Ⅰ |
| BLS-2 | Ⅰ |

Table S21 R language source code

install.packages('pheatmap')# Install package, load data

library(pheatmap) # Load the R package of pheatmap

# 1, Read heat map data file

df = read.delim("D:/heatmapchen/data.heatmap.txt", # Pay attention to file name, file path, and format

header = T, # Is there a title

sep = "\t", # The separator is the Tab key

row.names = 1, # Specify that the first column is the row name

fill=T) # Whether to automatically fill, generally select Yes

# (Optional) Read grouped data file

dfSample=read.delim("D:/heatmapchen/sample.class.txt",header=T,row.names=1,fill=T,sep ="\t")

dfGene=read.delim("D:/heatmapchen/gene.class.txt",header=T,row.names=1,fill=T,sep="\t")

# 2, draw

pheatmap(df,annotation_row=dfGene,annotation_col=dfSample,show_colnames=TRUE,show_rownames=TRUE,fontsize=6,color=colorRampPalette(c('#0000ff','#ffffff','#ff0000'))(50),annotation_legend=TRUE,border_color=NA,scale="row",cluster_rows=TRUE,cluster_cols=TRUE,clustering_distance_rows="manhattan",clustering_distance_cols="manhattan",clustering_method="mcquitty")

Remarks:

The clustering criteria for rows or columns can be selected：'correlation', 'euclidean', 'maximum', 'manhattan', 'canberra', 'binary', 'minkowski'。

clustering_method Optional parameters include 'ward.D', 'ward.D2', 'single', 'complete', 'average', 'mcquitty', 'median', 'centroid'。
